# Consensus-based recommendations on physical activity and exercise in patients with diabetes at risk of foot ulcerations: a Delphi study

**DOI:** 10.1016/j.bjpt.2023.100500

**Published:** 2023-04-06

**Authors:** Alba Gracia-Sánchez, Adriana López-Pineda, José Luis Lázaro-Martínez, Antonio Pérez, Francisco J. Pomares-Gómez, Lourdes María Fernández-Seguín, Vicente F. Gil-Guillén, Esther Chicharro-Luna

**Affiliations:** a Nursing Service, Department of Health and Behavioral Sciences, Faculty of Medicine, University Miguel Hernández de Elche, San Juan de Alicante, Spain; b Clinical Medicine Department, University Miguel Hernández de Elche, San Juan de Alicante, Spain; c Atenea Research Group, Foundation for the Promotion of Health and Biomedical Research, San Juan de Alicante, Spain; d Network for Research on Chronicity, Primary Care, and Health Promotion (RICAPPS), Alicante, Spain; e Diabetic Foot Unit,Universidad Complutense de Madrid. Instituto de Investigación Sanitaria del Hospital Clínico San Carlos (IdISSC), 28040 Madrid, Spain; f Endocrinology and Nutrition Department, Hospital de la Santa Creu i Sant Pau, Barcelona, Medicine Department, Universitat Autònoma de Barcelona, CIBER de Diabetes y Enfermedades metabólicas (CIBERDEM), Barcelona, Spain; g Endocrinology and Nutrition, University Hospital San juan de Alicante, San Juan de Alicante, Spain; h Physical Therapy Department, University of Seville, Seville, Spain; i Research Unit, University General Hospital of Elda, Elda, Spain

**Keywords:** Diabetic foot, Diabetic neuropathies, Exercise, Exercise therapy, Physical activity

## Abstract

**Background:**

Patients with diabetes mellitus are exposed to important complications. Currently little evidence exist on the guidelines that these patients, at some risk of foot ulceration, should follow for physical exercise.

**Objectives:**

To reach a consensus among multidisciplinary and international experts on physical activity/exercise recommendations for patients with diabetes, according to foot ulcer risk.

**Methods:**

Using a three-round Delphi method, a panel composed of 28 multidisciplinary experts in the management of diabetic foot assessed 109 recommendations on physical activity/exercise for patients with diabetes mellitus, according to their risk of foot ulcer. Consensus was assumed when 80% of responses matched the same category (agreement/disagreement).

**Results:**

Twenty-nine experts participated in the first and second rounds of consultation, and twenty-eight did so in the third, reaching final agreement on 86 of the 109 recommendations considered (78.9%). The study, thus, generated a consensus set of recommendations concerning different aspects of diabetic footcare before, during, and after exercise (e.g. when to examine the foot, how to assess it, what type of sock and insole to use, what types of exercise to perform, and when it is advisable to return to activity after an ulceration).

**Conclusion:**

This Delphi study generated recommendations based on the consensus of international experts on physical activity and exercise by patient with diabetes at risk of ulceration. Recommendations considered the state of the foot and the patient's history and status before physical activity and included information on intensity, duration, frequency, and progressions of physical activity/exercise, and the use of custom-made plantar orthoses, shoe prescription, and the convenience of returning to physical activity after an ulceration.

## Highlights

• We propose a series of structured recommendations, based on the consensus of international, multidisciplinary experts, on factors to consider before, during, and after physical activity/exercise, thus facilitating the advisory role of health professionals.
• These recommendations include the systematic analysis of the degree of risk presented by patients with diabetic foot before starting unsupervised physical activity.
• Patients at high risk of ulceration should be closely monitored, including during their return to activity after ulceration, and it is recommended that those with open ulcers or peripheral artery disease perform stretching and strengthening exercises.

## Introduction

According to the International Diabetes Federation (IDF), in 2019, 135.6 million people aged 65 years or more had diabetes, equivalent to 19.3% of the total population with diabetes worldwide. Moreover, if present trends continue, the number of people aged 65 years or over with diabetes will rise to 195.2 million by 2030 and 276.2 million by 2045.^1^

People with diabetes mellitus (DM) are at increased risk of developing serious health problems that could lead to a poorer quality of life and increase healthcare costs.^2^ One such problem is foot ulcers, which are associated with increased morbidity, mortality, and healthcare costs.^3^^,^^4^ It has been estimated that 15–25% of people with diabetes will be affected by a foot ulcer at some point in their lives.^5^^,^^6^

Diabetes induces characteristic pathological changes in the feet, such as infection, diabetic foot ulcer, and neuroarthropathy, broadly termed diabetic foot syndrome.^7^ This condition is normally addressed by preventive strategies including patient education, risk stratification, and regular foot evaluations for peripheral vascular disease and neuropathy.^8^ In addition, physical activity and exercise enhance glycemic control and nerve function for people with DM, hence reducing the risk of diabetic neuropathy, a major risk factor for foot ulcers.

Physical activity is defined as any movement that increases energy use, as distinct from exercise, which is a planned and structured physical activity. Physical activity improves blood glucose control in type 2 DM, reduces cardiovascular risk, contributes to weight loss, and enhances well-being.^9^^,^^10^ If performed regularly, it can prevent or delay the development of type 2 DM.^11^

Although physical activity and exercise are recommended for people with DM,^12^, ^13^, ^14^ information is scarce on the type of activity most suitable for people at risk of developing foot ulcers (e.g., those with neuropathy, peripheral arterial disease [PAD], or foot deformities) and on the specific impact made by physical activity on the foot and on the risk of ulceration.

Patients with diabetes with impaired protective sensitivity and restricted pain response are vulnerable to trauma and extrinsic forces from ill-fitting footwear. Motor neuropathy causes muscle weakness and an intrinsic muscle imbalance that can lead to deformities such as hammer or claw toes, which in turn create elevated plantar pressure due to instability of the metatarsophalangeal joint. For this reason, performing physical activity or exercise without medical supervision can create a risk for persons with DM.^15^

In clinical practice, uncertainty remains about the best type and intensity of exercise for these patients, especially those at high risk of developing a foot ulceration or re-ulceration. Accordingly, the aim of this study was to reach a consensus among multidisciplinary and international experts on recommendations for physical activity/exercise by people with DM, according to their risk of foot ulcers.

## Methods

### Study design

This observational, cross-sectional, descriptive study was conducted using a three-round Delphi technique,^16^ that is commonly used to identify areas of agreement among experts in diverse areas of knowledge^17^, ^18^, ^19^ in the absence of full agreement or when knowledge is incomplete, uncertain or unproven.^20^ The Delphi method offers several advantages over other consensus techniques such as focus groups or nominal groups. First, it does not require the physical presence of the experts, enabling them to participate at a distance in space and time. In addition, the anonymity provided fosters free expression and minimizes the influence of opinion leaders. Specifically, the present study was conducted following the CREDES guide (Conducting and REporting of DElphi Studies),^21^ details of which have been published previously.^22^ This study was approved by the Office for Responsible Research at our institution. No authorization from the ethics committee was required, as no personal patient data were used.

### Participants

Four experts working in Spain in the field of diabetic foot (two endocrinologists, a podiatrist, and a physical therapist) were recruited to the scientific committee. These experts, selected out of convenience from the research group's professional network, conducted the critical review of the first version of the Delphi survey created by the research team, reviewed the selection criteria for the expert panel, and identified other experts to participate in the study.

An expert panel was formed to elicit the opinions of international, multidisciplinary experts working in various fields related to the management of diabetic foot (endocrinology, family medicine, vascular surgery, podiatry, physical therapy, and physical activity and sports), through an online Delphi survey to be completed individually and anonymously.

There are no firm recommendations regarding the optimal size of a Delphi panel in heterogeneous samples. In recent Delphi studies, some panels have included as many as 60 members,^17^^,^^23^ while others have had 40,^24^ or as few as 19.^18^ In our study, invitations were emailed to 60 professionals with clinical and scientific experience in diabetes and who met the study selection criteria (these are specified in detail in the study protocol). Twenty experts were initially selected from each group.^22^ The panelists were not required to be physically present, but only to respond online to the Delphi survey questions, within the stipulated period.

### Delphi method

The research group developed an adapted version of the Delphi survey based on a literature review of guidelines related to physical activity and diabetes^14^^,^^25^, ^26^, ^27^, ^28^, ^29^, ^30^, ^31^, ^32^, ^33^, ^34^, ^35^, ^36^, ^37^, ^38^, ^39^ The scientific committee then reviewed and modified this draft survey to create a final version. Foot risk was stratified in accordance with the guidelines published by the International Working Group on the Diabetic Foot (IWGD), as follows: risk 0 (very low risk): no loss of protective sensation (LOPS) and no PAD; IWGDF risk 1 (low risk): LOPS or PAD; IWGDF risk 2 (moderate risk): LOPS + PAD, or LOPS + foot deformity, or PAD + foot deformity; IWGDF risk 3 (high risk): LOPS or PAD, and one or more of the following; history of foot ulcer, a lower-extremity amputation (minor or major), end-stage renal disease.^12^ The final Delphi survey consisted of 50 statements, with a total of 109 items, grouped into recommendations for before, during, and after physical activity/exercise (Supplementary Material 1). A pilot study was conducted with 10 of the panelists to ensure that the survey items were readily comprehensible and that its method of application was appropriate.

A letter of invitation, following the script proposed by Jon Landeta,^40^ was sent by email to the professionals selected for the expert panel. This letter included a link to access the online survey created using Google Forms. The professionals who accepted this invitation were asked to rate their level of agreement or disagreement with each survey item on a 5-point Likert scale, ranging from 1 = total disagreement, to 3 = neither agreement nor disagreement, and 5 = total agreement. A free text space was also provided for each survey item, where the panelist could make any comment considered appropriate.

The Delphi process consisted of three rounds. After performing the pilot study, which proceeded without suggestions being offered, the first round commenced on 12 June 2021. The panelists were given eight weeks to complete the survey, during which time three reminders were sent, if necessary, to maximize the response rate. After the first round, the researcher responsible for maintaining the participants’ confidentiality downloaded the data into an Excel sheet, which was then analyzed by the entire team. The second and third rounds began on 19 September and 23 October 2021, respectively, inviting responses from the experts who had participated in the previous rounds. In this case the panelists were given two weeks to respond to each round, which included only the items that had not previously produced a consensus, and to provide, if they wished, explanatory comments in the free-form text fields. The final list of recommendations was based on the items for which a consensus was reached.

### Statistical analysis

The panelists’ specialties and countries were characterized by absolute and relative frequencies. Their responses to each survey item were analyzed in terms of the percentage of response categories obtained, as is the case in most of the studies cited in the CREDES guide.^21^ Ratings of 4 or 5 were considered to indicate “agreement” and of 1 or 2 “disagreement.” When a response category was agreed upon by 80% or more of the panelists, the statement was considered to have obtained consensus,^41^ and it was omitted from the following round of consultation. All analyses were performed using the Microsoft Excel program (Microsoft, Redmond, WA, USA).

## Results

**Fig. 1:**
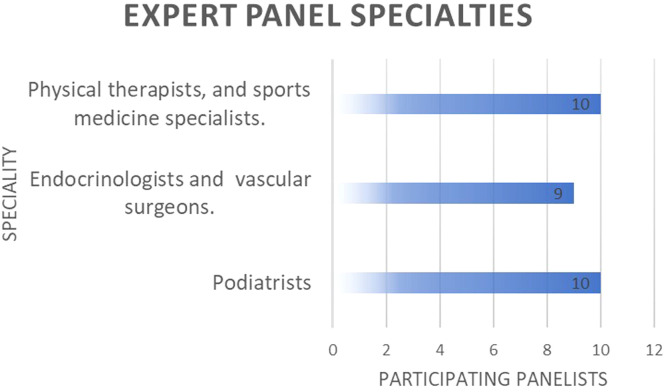
Specialties of expert panelists.

In the first round, 29 experts (48.3% response rate) responded to the Delphi survey. By specialties, 10 of these participants were experts in physical activity, sports sciences, or physical therapy, 10 were podiatrists and 9 were endocrinologists, family physicians, or vascular surgeons (Fig. 1). Most were of Spanish nationality, but a significant number of panelists from other countries also took part (Table 1).

**Table 1:** Nationalities of expert panelists.

Country	No. panelists
United States	2
Canada	1
Italy	3
Spain	16
Chile	1
United Kingdom	2
Netherlands	1
Portugal	2
Dominican Republic	1

In no case did disagreement among the panelists reach 80%, and so none of the items were directly eliminated. The results of the first round are provided in Supplementary Material 2 – S.1. This round took 73 days due to provided extension time. Consensus was reached on 42 items (38.5%), and the remaining 67 items were included in the second round of the survey. After reviewing the panelists’ comments, some items were reformulated or qualified, but there were no fundamental changes to the original meaning (the modifications are detailed in Supplementary Material 2 – S.2). The research team drafted a report on the round 1 results, which was sent to the panelists together with the round 2 survey.

The second round, which took 24 days, during which the panelists received two reminders, achieved a response rate of 100% (*n* = 29) (Supplementary Material 2 – S.3). A consensus was reached on another 34 of the survey items (31.2%), leaving 33 items to be repeated or reformulated in the third round.

For round 3, which lasted 41 days the response rate was 96.6% (*n* = 28). The initial deadline was extended, and four reminders were sent to maximize the number of participants. Once the responses were recorded, the final analysis for round 3 was performed (Supplementary Material 2 – S.4). In this round, consensus was reached on 10 additional items (9.2%).

By the end of the third round, the panelists had reached a consensus on 86 items (78.9%) (Table 2). The 23 items (21.1%) for which no consensus was obtained were removed from the final recommendations (Supplementary Material 2 – S.5). These results formed the basis for a final table of recommendations for patients with diabetes for before, during, and after exercise, according to the foot risk presented (Table 3). Pre-exercise recommendations included questions such as when and how to inspect the foot, the initial medical examination of the patient's general condition, the type of sock recommended for patients with PAD, the impact of neuropathy and/or foot deformities on the risk of foot ulcers (IWGDF 0–3), and the most appropriate type of exercise, taking into account its intensity, duration, frequency and progression. Recommendations on aspects to consider during and after exercise/physical activity included the use of custom-made plantar orthoses, the prescription of footwear, the types of exercise to perform, and the timetable for a return to activity after an ulceration.

**Table 2:** Delphi survey items that reached consensus, by consultation round.

Recommendations prior to commencing physical activity: General recommendations for people at any IWGDF risk (categories 0–3) prior to commencing physical activity	Agree 1ST Round	Agree 2ST Round	Agree 3ST Round
**1. The patient themselves should inspect both feet before beginning physical activity, checking for:**			
1.1 Moisturization of the feet, assessing potential dryness of the forefoot, heels, or other areas	82.8%		
1.3 Adequate length of the toenails	89.7%		
1.4 Sharp edges on toenails	93.1%		
1.5 Presence of hyperkeratosis or calluses	93.1%		
1.6 Presence of blisters	96.6%		
1.7 Presence of wounds	100%		
1.8 Presence of irregularities, wear and tear, or other alterations on the inside of footwear	93.1%		
**2. An initial examination of the patient's general condition should be undertaken, including an assessment of:**			
2.1 Body Mass Index (BMI). Recommended activity levels should take into account the presence of obesity	72.4%	86.2%	
2.2 Age	82.8%		
2.3 Possible cardiovascular alterations	93.1%		
2.4 Evaluation of limited joint mobility	93.1%		
2.5 Existence of arterial hypertension	89.7%		
2.6 The presence of uncontrolled retinopathy or renal impairment may influence the type of exercise recommended	79.3%	100%	
**3. With regard to the diabetes, the attending physician or healthcare provider should assess the type (1 or 2), the level of metabolic control, and the treatment in order to determine the risk of hypoglycemia during physical activity**	96.6%		
**4. People under treatment with insulin and/or sulfonylureas:**			
4.1 Should take a glucose reading before exercising	82.8%		
4.2 Glucose levels should be monitored during exercise, and more frequently for high-risk patients being treated with insulin	65.5%	82.7%	
4.3 Should take a glucose reading after exercising	82.8%		
4.4 If necessary, dietary and/or pharmacological adjustments should be recommended by the healthcare provider, and a glycogen kit should be available	72.4%	86.2%	
**5. The presence of keratotic lesions or excessive dryness on different areas of the foot:**			
5.1 These conditions should be treated before establishing the intensity and duration of the exercise recommended	69.0%	86.2%	
5.2 These conditions should be treated before establishing the type of exercise recommended	79.3%	93.1%	
**6. The type of sock recommended should be conditioned by:**			
6.1 The presence of complications like neuropathy, foot deformities, or peripheral artery disease (PAD)	82.8%		
6.2. The type of sock to be recommended should be specific, according to the physical activity to be carried out, but preferably light-coloured to visualize any bleeding more easily, clean, seamless and with no tight-fitting elastics	65.5%	68.9%	89.3%
**7. In the case of PAD (peripheral artery disease), the patient should use:**			
7.2 Socks without seams, rubber, or elastic that could compromise circulation	89.7%		
**8. In case of neuropathy, patients should use:**			
8.3 Socks without seams, rubber, or elastic	89.7%		
**9. A professional should always be consulted about the type of footwear before the patient begins an exercise regimen**	79.3%	93.1%	
**10. The type of exercise recommended should take into account the patient's preferences**	93.1%		
**11. Group exercise activities with people presenting similar risk factors can help motivate the patient to engage in regular physical activity**	37.9%	75.8%	89.3%
**12. The use of a smart watch or mobile apps during exercise is advised in patients with high cardiovascular risk in order to monitor:**			
12.1 Pulse	82.8%		
12.2 Blood pressure	72.4%	82.8%	
12.3 Intensity of physical activity	69%	89.7%	
12.4 Type and duration of physical activity	72.4%	93.1%	
**13. Patients at risk of HYPOGLYCEMIA should:**			
13.1 Take fast-acting carbohydrates with them during exercise sessions	93.1%		
13.3 It is important to drink plenty of liquid during exercise, to promote hydration.	79.3%	93.1%	
**14. Regarding the characteristics of the exercise, it should be progressive, with moderate intensity in the first sessions and gradually becoming more vigorous according to the patient's circumstances and ability**	93.1%		
**15. In case of water activities, patients should use preventive measures to avoid foot infections**	82.8%		
**Specific recommendations prior to physical activity for people with iwgdf risk 1, 2, and 3:**			
**16. In patients with IWGDF risk 1, 2, or 3, the presence of any keratotic lesions or blisters should preclude any physical activity pending consultation with a healthcare professional**	82.8%		
**17. Patients with hyperkeratosis, prior amputations, or calluses on the sole (IWGDF 2 or 3) should wear therapeutic footwear, including custom-made shoes, and receive orthopedic/podiatric treatment to redistribute areas of hyperpressure before doing any exercise**	96.6%		
**18. Patients with IWGDF 2 or 3 should inspect their feet:**			
18.1 Before exercise	100%		
18.2 During any prolonged physical exercise (exceeding one hour), it is advisable to examine the feet (without socks) for possible injury	58.6%	62%	89.2%
18.3 On finishing exercise	100%		
18.4 Foot inspections should consider temperature, color, and signs of lesions	96.6%		
18.5 Sensations such as pain, paraesthesia, or itching should be taken into account	75.9%	96.6%	
**19. In case of NEUROPATHY, patients should consult a podiatrist for:**			
19.1 A biomechanical study and orthopedic/podiatric treatment if appropriate	100%		
19.2 At IWGDF 2, biomechanical footwear should be prescribed	69.0%	89.7%	
19.3 At IWGDF 3, biomechanical and/or custom-made footwear should be prescribed	65.5%	96.6%	
**Specific recommendations prior to physical activity for people with IWGDF risk 3:**			
**21. If the ulcer had a plantar location and has completely healed (the wound has not opened for 15 days after epithelialization), before beginning exercise, the patient should:**			
21.1 Receive personalized orthopedic/podiatric treatment	82.8%		
21.2 Patients should use biomechanical footwear and/or footwear adapted to individual characteristics (such as high toe or special width)	62.1%	96.6%	
21.3 In case of amputation, the stump should be checked to ensure an even distribution in the pressure zones before supporting a load	96.6%		

**Type of exercise: intensity, duration, frequency, progression:** General recommendations for people at any IWGDF risk (categories 0–3) regarding the type of exercise: intensity, duration, frequency, progression.			

**23. Patients should be encouraged to perform aerobic activities as well as strength training and stretching, adapted to each type of at-risk foot**	93.1%		
24. In patients receiving rehabilitation treatment, telemonitoring facilitates the control of adherence to the exercise program	75.9%	86.2%	
25. The use of telemonitoring can improve patients’ adherence to physical exercise programmes, whatever the degree of foot risk	72.4%	86.2%	
**26. All programmed sessions of physical activity should include:**			
26.1 A warm-up prior to exercise of at least 5 min	89.7%		
26.2 Gentle stretching prior to commencing the exercise	82.8%		
26.3 On finishing the exercise, a cool-down of at least 5 min of slow walking	79.3%	82.8%	
**27. Patients with neuropathy (IWGDF 1, 2 and 3) should:**			
27.1 Do exercises to improve static balance	55.2%	82.8%	
27.2 Do exercises to improve dynamic balance	62.1%	86.2%	
27.3 Begin with low- or moderate-intensity aerobic exercise, appropriate to the patient's age and physical characteristics	86.2%		
**28. If the patient has suffered motor neuropathy, regardless of the degree of risk, rehabilitation exercises should be performed to limit the progression of the deformities**	48.3%	69.0%	85.7%
**29. In patients with mild to moderate PAD (IWGDF 1, 2, or 3), the healthcare provider should consider adding specific exercises to improve vascular function.**	75.9%	86.2%	
**Specific recommendations for patients with IWGDF 0, according to the type of exercise: intensity, duration, frequency, progression**			
**30. The patient should be encouraged to do as much physical activity as possible (walking up the stairs instead of using the elevator, walking to shops instead of driving, etc.)**	96.6%		
**31. In the absence of any cardiovascular alterations, the patient should:**			
31.1 Do aerobic activity	89.7%		
31.2. Each exercise session should consist of 30–60 min of aerobic exercise	75.9%	75.9%	82.1%
**32. Exercise activities should include strength training: from passive movements of the ankle joint to active resistance (using a band) to work the ankle (dorsi and plantar flexion), the forefoot (inversion-eversion) and the toes (flexion-extension, abduction-adduction) at least twice a week**	72.4%	82.8%	
**Specific recommendations for patients with IWGDF 1. according to the type of exercise: intensity, duration, frequency, progression**			
**33. Patients with peripheral neuropathy should:**			
33.1 Patients should walk (at a moderate pace) for one hour, three times a week, starting with 30 min per session and progressively increasing the duration	75.9%	82.8%	
33.2 Muscle strengthening exercises, with active movements against resistance, should be performed at least twice a week	69%	89.7%	
33.3 In case of coexisting obesity, do aerobic activity that does not overload joints or put excessive pressure on the feet (cycling, swimming)	79.3%	93.1%	
33.4. If the patient rides a bicycle, in addition to recommending a specific orthosis within the footwear according to the biomechanical alteration presented, a discharge device could be added to the pedal area to help redistribute the pressure if this area presents hyperkeratosis	65.5%	69.0%	82.1%
**34. Patients with mild to moderate PAD (Rutherford grade 1 or 2) WITHOUT NEUROPATHY should begin walking at a moderate intensity and gradually increase it based on the onset of pain due to intermittent claudication.**	79.3%	82.8%	
**Specific recommendations for patients with IWGDF 2, according to the type of exercise: intensity, duration, frequency, progression**			
**35. Patients with IWGDF 2, neuropathy and foot deformity should:**			
35.1 Low-intensity aerobic exercise is recommended, initially for 5–10 min and progressively increasing to a duration of 25–30 min per day during the week on alternate days	65.5%	75.1%	85.7%
35.2 If the patient rides a bicycle, in addition to recommending a specific orthosis within the footwear according to the biomechanical alteration presented, a discharge device could be added to the pedal area to help redistribute the pressure if this area presents hyperkeratosis	72.4%	75.9%	82.1%
35.3 Add range-of-motion exercises: passive movements to the extent possible in the ankle joints (dorsi and plantar flexion), the forefoot (inversion-eversion) and the toes (flexion-extension, abduction-adduction) at least twice a week	72.4%	93.1%	
**36. For patients with Grade 2-foot risk, with neuropathy and deformity, the aerobic exercise of walking can be replaced by aquatic activities, producing no pressure on the foot, 2–3 times a week. These patients should always use appropriate footwear and maintain good foot hygiene**	62.1%	72.4%	85.7%
**Specific recommendations for patients with IWGDF 3, according to the type of exercise: intensity, duration, frequency, progression**			
**38. The healthcare provider should assess the patient's routine physical activity, eliminating or modifying some activities if the patient presents lengthy, unregulated walking or physical efforts in excess of their level of tolerance**	89.7%		
**39. Patients with open ulcer should not put any pressure on the lesion or perform any exercises that put weight on the area where the ulcer is located**	93.1%		
**40. Patients with IWGDF 3 and an open ulcer without PAD should:**			
40.1 Stretching and strength-training exercises should be performed, in a seated or supine position, according to the patient's characteristics and the location of the ulcer	79.3%	96.6%	
40.2 Work on ankle mobility, plantar flexion, dorsiflexion, inversion, eversion, circumduction, and dorsi and plantar flexion of the toes at least 3 times a week or every other day, adapting all activities to the patient's characteristics	69.0%	82.8%	
**41. Patients with severe PAD (with lesions) should:**			
41.1 Do mobility exercises of the lower limbs in a seated or supine position	86.2%		
41.2 Exercises to improve ankle mobility, plantar flexion, dorsiflexion, inversion, eversion, circumduction and plantar and dorsi flexion of the toes should be performed three times per week or every other day, provided it does not provoke pain or aggravate the injury	79.3%	93.1%	
41.3 Adapt all exercise sets to the symptomology related to the lesion and its location	86.2%		
**42. Patients with IWGDF 3 and a recent (<15 days) history of ulceration should:**			
42.1. The activities of daily life should be started, with at least 10 minutes’ activity every day using a plantar orthosis and technical assistance (such as a cane or crutch) and gradually progressing over the next 15 days, according to the patient's physical condition	58.6%	72.4%	82.1%
42.2 Initially, the patient should perform at least 10 min activity per day wearing a plantar orthosis and gradually increase the duration over the next 15 days if possible.	69.0%	86.2%	
**44. Patients with IWGDF 3 and a prior amputation should consult a rehabilitation specialist to strengthen the stump and treat the phantom limb with functional exercises.**	86.2%		

**Recommendations during exercise:** Specific recommendations for patients with IWGDF 1. 2. or 3			

**46. Patients with neuropathy and foot deformity should consider using an assistive device such as a cane or crutch, if their balance is significantly disturbed**	79.3%	96.6%	

**General recommendations after exercise for patients with IWGDF risk**			

**48. Risk factors for ulceration in patients with all categories of IWGDF risk who are performing moderate- to high-intensity physical activity should be monitored at least as frequently as recommended by the IWGDF: IWGDF 0: once yearly; IWGDF 1: every 6 to 12 months; IWGDF 2: every 3 to 6 months; IWGDF 3: every 1 to 3 months. Examinations should also include a review of the footwear being used during physical activity exercises**	86.2%		
**49. Healthcare providers should follow up patients with IWGDF 2 and 3 every month to monitor the recommended exercise regimen and modify it according to the patient's evolution**	79.3%	93.1%	
**50. Any patient with diabetes and neuropathy, or who uses a custom-made plantar orthosis, should be examined 15 days after starting physical exercise by the healthcare provider. This examination should be repeated periodically to detect any wear or irregularities in the material after its use in physical exercise**	75.9%	93.1%	

Bold type: main statement; grey table cell: round consensus item; black table cell: title thematic blocks.

**Table 3:** List of recommendations by IWGDF risk.

Recommendations prior to commencing physical activity for people at any IWGDF risk (categories 0–3)				
**1. The patient themselves should inspect both feet before beginning physical activity, checking for:** 1.1 Moisturization, assessing potential dryness of the forefoot, heels, or other areas. (82.8%) 1.3 Adequate length of the toenails (89.7%) 1.4 Sharp edges on toenails (93.1%) 1.5 Presence of hyperkeratosis or calluses (93.1%) 1.6 Presence of blisters (96.6%) 1.7 Presence of wounds (100.0%) 1.8 Presence of irregularities, wear and tear, or other alterations on the inside of footwear (93.1%)				
**2. An initial examination of the patient's general condition, not foot-specific, should be undertaken, including an assessment of:** 2.1 Body mass index (BMI). recommended activity levels should take into account the presence of obesity (86.2%) 2.2 Age (82.8%) 2.3 Possible cardiovascular alterations (93.1%) 2.4 Evaluation of limited joint mobility (93.1%) 2.5 Existence of arterial hypertension (89.7%) 2.6 The presence of uncontrolled retinopathy or renal impairment may influence the type of exercise recommended (100%)				
**3. With regard to the diabetes, the attending physician or healthcare provider should assess the type (1 or 2), the level of metabolic control, and the treatment to determine the risk of hypoglycemia during physical activity (96.6%)**				
**4. People under treatment with insulin and/or sulfonylureas:** 4.1 Should take a glucose reading before exercising (82.8%) 4.2 Glucose levels should be monitored during exercise more frequently in high-risk patients being treated with insulin (82.7%) 4.3 Should take a glucose reading after exercising (82.8%) 4.4 If necessary, the healthcare provider should recommend dietary and/or pharmacological adjustments, and a glycogen kit should be available (86.2%)				
**5. The presence of keratotic lesions or excessive dryness on different areas of the foot:** 5.1 These conditions should be treated before establishing the intensity and duration of the exercise recommended (86.2%) 5.2 These conditions should be treated before establishing the type of exercise recommended (93.1%)				
**6. The type of sock recommended should be conditioned by:** 6.1 The presence of complications like neuropathy, foot deformities, or peripheral arterial disease (PAD) (82.8%) 6.2 The type of sock to be recommended should be specific, according to the physical activity to be carried out, but preferably light-colored (to visualize any bleeding more easily), clean, seamless, and with no tight-fitting elastics (89.3%)				
**7. In the case of PAD (peripheral arterial disease), the patient should use:** 7.2 Socks without seams, rubber, or elastic that could compromise circulation (89.7%)				
**8. In case of neuropathy, patients should use:** 8.3 Socks without seams, rubber, or elastic (89.7%)				
**9. A PROFESSIONAL should always be consulted about the type of footwear before the patient begins an exercise regimen (93.1%)**				
**10. The type of exercise recommended should take into account the patient's preferences (93.1%)**				
**11. Group exercise activities with people presenting similar risk factors can help motivate the patient to engage in regular physical activity (89.3%)**				
**12. The use of a smart watch or mobile apps during exercise is advised in patients with high cardiovascular risk to monitor:** 12.1 Pulse (82.8%) 12.2 Blood pressure (82.8%) 12.3 Intensity of physical activity (89.7%) 12.4 Type and duration of physical activity (93.1%)				
**13. Patients at risk of HYPOGLYCEMIA should:** 13.1 Take fast-acting carbohydrates with them during exercise sessions (93.1%) 13.3 It is important to drink plenty of liquid during exercise to promote hydration (93.1%)				
**14. Regarding the characteristics of the exercise, it should be progressive, with moderate intensity in the first sessions and gradually becoming more vigorous according to the patient's circumstances and ability (93.1%)**				
**15. In case of water activities, patients should use preventive measures to avoid foot infections (82.8%)**				

**Specific recommendations prior to physical activity for people with IWGDF risk**	**IWGDF risk 0**	**IWGDF risk 1**	**IWGDF risk 2**	**IWGDF risk 3**

**16. In patients with IWGDF risk 1, 2, or 3, the presence of any keratotic lesions or blisters should preclude any physical activity pending consultation with a healthcare professional (82.8%)**		X	X	X
**17. Patients with hyperkeratosis, prior amputations, or calluses on the sole (IWGDF 2 or 3) should wear therapeutic footwear, including custom-made shoes, and receive orthopedic/podiatric treatment to redistribute areas of hyperpressure before doing any exercise (96.6%)**			X	X
**18. Patients with IWGDF 2 or 3 should inspect their feet:** 18.1 Before exercise (100%) 18.2. During any prolonged physical exercise (exceeding one hour), examining the feet (without socks) for possible injury (89.3%) 18.3 On finishing exercise (100%) 18.4 Considering temperature, color, and signs of lesions (96.6%) 18.5 Considering sensations such as pain, paresthesia, or itching (95.6%)			X	X
**19. In case of neuropathy, patients should consult a podiatrist for:** 19.1 A biomechanical study and orthopedic/podiatric treatment if appropriate (100%) 19.2 At IWGDF 2, a prescription for biomechanical footwear (89.7%) 19.3 At IWGDF 3, a prescription for biomechanical and/or custom-made footwear (96.6%)		X	X	X
**21. If the ulcer had a plantar location and has completely healed (the wound has not opened for 15 days after epithelialization), before beginning exercise, the patient should:** 21.1 Receive personalized orthopedic/podiatric treatment (82.8%) 21.2 Use biomechanical footwear and/or footwear adapted to individual characteristics (such as high toe or special width) (96.6%) 21.3 In case of amputation, check the stump to ensure an even distribution in the pressure zones before supporting a load (96.6%)				X

**Specific recommendations during and after exercise for patients with IWGDF risk**	**IWGDF risk 0**	**IWGDF risk 1**	**IWGDF risk 2**	**IWGDF risk 3**

**46. Patients with neuropathy and foot deformity should consider using an assistive device, such as a cane or crutch, if their balance is significantly impaired (96.6%)**			X	X
**48. Risk factors for ulceration in patients with all categories of IWGDF risk who are performing moderate- to high-intensity physical activity should be monitored at least as frequently as recommended by the IWGDF: IWGDF 0, once yearly; IWGDF 1, every 6 to 12 months; IWGDF 2, every 3 to 6 months; IWGDF 3, every 1 to 3 months. Examinations should also include a review of the footwear being used during physical activity exercises (86.2%)**	X	X	X	X
**49. Healthcare providers should follow up patients with IWGDF 2 and 3 every month to monitor the recommended exercise regimen and modify it according to the patient's evolution (93.1%)**			X	X
**50. Any patient with diabetes and neuropathy, or who uses a custom-made plantar orthosis, should be examined 15 days after starting physical exercise by the healthcare provider. This examination should be repeated periodically to detect any wear or irregularities in the material after its use in physical exercise (93.1%)**			X	X
Recordatory: 18.3 Patients with IWGDF 2 or 3 should inspect their feet on finishing exercise. (100%)			X	X

**General recommendations on type of exercise for people at any IWGDF risk (categories 0–3)**				

**23. Patients should be encouraged to perform aerobic activities as well as strength training and stretching, adapted to each type of at-risk foot (93.1%)**				
**24. In patients receiving rehabilitation treatment, telemonitoring facilitates the control of adherence to the exercise program (86.2%)**				
**25. The use of telemonitoring can improve patients’ adherence to physical exercise programs, whatever the degree of foot risk (86.2%)**				
**26. All programmed sessions of physical activity should include:** 26.1 A warm-up prior to exercise of at least 5 min (89.7%) 26.2 Gentle stretching prior to commencing the exercise (82.8%) 26.3 On finishing the exercise, a cool-down of at least 5 min of slow walking (82.8%)				

**Specific recommendations for people at any IWGDF risk (categories 0–3)** **Type of exercise: intensity, duration, frequency, progression**	**IWGDF risk 0**	**IWGDF risk 1**	**IWGDF risk 2**	**IWGDF risk 3**
**27. Patients with neuropathy (IWGDF 1, 2 and 3) should:** 27.1 Do exercises to improve static balance (82.8%) 27.2 Do exercises to improve dynamic balance (86.2%) 27.3 Begin with low- or moderate-intensity aerobic exercise, appropriate to the patient's age and physical characteristics (86.2%)		X	X	
**28. If the patient has suffered motor neuropathy, regardless of the degree of risk, rehabilitation exercises should be performed to limit the progression of the deformities (85.7%)**		X	X	
**29. In patients with mild to moderate PAD (IWGDF 1, 2, or 3), the healthcare provider should consider adding specific exercises to improve vascular function (86.2%)**		X	X	
**30. The patient should be encouraged to do as much physical activity as possible (walking up the stairs instead of using the elevator, walking to shops instead of driving, etc.) (96.6%)**	X			
**31. In the absence of any cardiovascular alterations:** 31.1 The patient should do aerobic activity (89.7%) 31.2. Each exercise session should consist of 30–60 min of aerobic exercise (82.1%)	X			
**32. Exercise activities should include strength training: from passive movements of the ankle joint to active resistance (using a band) to work the ankle (dorsi and plantar flexion), the forefoot (inversion-eversion) and the toes (flexion-extension, abduction-adduction) at least twice a week (82.8%)**	X			
**33. Patients with peripheral neuropathy should:** 33.1 Walk (at a moderate pace) for one hour, three times a week, starting with 30 min per session and progressively increasing the duration (82.8%) 33.2 Perform muscle strengthening exercises, with active movements against resistance, at least twice a week (89.7%) 33.3 In case of coexisting obesity, do aerobic activity that does not overload joints or put excessive pressure on the feet (cycling, swimming) (93.1%) 33.4. In case of riding a bicycle, be advised to use a specific orthosis within the footwear according to the biomechanical alteration presented and to use an offloading device in the pedal area to help redistribute the pressure if this area presents hyperkeratosis (82.1%)		X	X	
**34. Patients with mild to moderate PAD (Rutherford grade 1 or 2) without neuropathy should begin walking at a moderate intensity and gradually increase it based on the onset of pain due to intermittent claudication (84.8%)**		x		
**35. Patients with neuropathy and foot deformity should:** 35.1 Do low-intensity aerobic exercise, initially for 5–10 min and progressively increasing to 25–30 min/day during the week on alternate days (85.7%) 35.2 In case of riding a bicycle, be advised to use a specific orthosis within the footwear according to the biomechanical alteration presented and to use an offloading device in the pedal area to help redistribute the pressure if this area presents hyperkeratosis (82.1%) 35.3 Add range-of-motion exercises: passive movements to the extent possible in the ankle joints (dorsi and plantar flexion), the forefoot (inversion-eversion) and the toes (flexion-extension, abduction-adduction) at least twice a week (93.1%)			x	
**36. For patients with IWGDF 2-foot risk, with neuropathy and deformity, the aerobic exercise of walking can be replaced by aquatic activities, producing no pressure on the foot, 2–3 times a week. These patients should always use appropriate footwear and maintain good foot hygiene (85.7%)**			x	
**38. The healthcare provider should assess the patient's routine physical activity, eliminating or modifying some activities if the patient presents lengthy, unregulated walking or physical efforts in excess of their level of tolerance (89.7%)**			X	
**39. Patients with an OPEN ULCER should not put any pressure on the lesion or perform any exercises that put weight on the area where the ulcer is located (93.1%)**				X
**40. Patients with IWGDF 3 and an open ulcer without PAD should:** 40.1 Perform stretching and strength-training exercises, in a seated or supine position, according to the patient's characteristics and the location of the ulcer (96.6%) 40.2 Work on ankle mobility, plantar flexion, dorsiflexion, inversion, eversion, circumduction, and dorsi and plantar flexion of the toes at least 3 times a week or every other day, adapting all activities to the patient's characteristics (82.8%)				X
**41. Patients with severe PAD (with lesions) should:** 41.1 Do mobility exercises of the lower limbs in a seated or supine position (86.2%) 41.2 Perform exercises to improve ankle mobility, plantar flexion, dorsi flexion, inversion, eversion, circumduction and plantar and dorsi flexion of the toes, three times per week or every other day, provided it does not provoke pain or aggravate the injury (93.1%) 41.3 Adapt all exercise sets to the symptomology related to the lesion and its location (86.2%)				X
**42. In patients with IWGDF 3 and a recent (<15 days) history of ulceration:** 42.1 The activities of daily life should be started, with at least 10 minutes’ activity every day, using a plantar orthosis and technical assistance (such as a cane or crutch) and gradually progressing over the next 15 days, according to the patient's physical condition (82.1%) 42.2 Initially, the patient should perform at least 10 minutes’ activity per day wearing a plantar orthosis and gradually increase the duration over the next 15 days, if possible (86.2%) 44. Patients with IWGDF 3 and a prior amputation should consult a rehabilitation specialist to strengthen the stump and treat the phantom limb with functional exercises (86.2%)				X

Bold type: main statement; Black table cell: title thematic blocks.

## Discussion

The experts’ consensus recommendations for patients with diabetic foot are summarized as follows. Before starting any physical activity, all patients should undergo a prior check-up of both feet, and those classified as risk 2 or 3 should repeat this check-up during and after the activity. In addition, every patient should receive a prior assessment of their general condition and of any keratotic lesions or excessive dryness in different areas of the foot.

When neuropathy and/or PAD are observed, the socks worn should have no seams, rubber, or elastic, and the choice of footwear should be guided by a professional. Aerobic exercise is recommended, with strength and flexibility training adapted to each type of foot at risk, such as the inclusion of rehabilitation exercises for patients with motor neuropathy or the practice of aquatic exercise rather than walking, thus avoiding pressure on the foot, for those at high risk of ulceration. Orthopedic treatment is recommended for patients at risk 3, while those at risk 2 or 3 should receive a check-up 15 days after starting physical exercise and monthly at the location where the exercise takes place.

These recommendations are consistent with those of the American Diabetes Association,^38^ stating that patients with type 2 diabetes should perform three or more sessions of moderate-to-vigorous-intensity physical activity per week, for a total of at least 150 min and avoid two or more consecutive days without physical activity/exercise. The latter recommendations, however, do not provide enough data on appropriate exercises for people at high risk of foot ulceration.

Shortly after the completion of this study, the American College of Sports Medicine published new recommendations, with clarifications regarding the types of exercise appropriate for patients with peripheral neuropathy, among other aspects also addressed in our study. These guidelines are consistent with the conclusions drawn from our study.^42^ However, these recommendations are not specifically adapted to different risk categories for diabetic foot.

The IWGDF^39^ recommends various forms of foot-related exercises, such as strengthening and stretching, to alleviate risk factors for the incidence of foot ulceration,^43^, ^44^, ^45^, ^46^ but their recommendations do not provide recommendation on frequency, and do not differentiate between foot risk categories. In the present study, more closely-targeted recommendations have been obtained, for example that patients with neuropathy (IWGDF risk 1, 2, or 3) should perform exercises to improve static and dynamic balance, beginning with low or moderate-intensity aerobic exercise appropriate to the patient's age and physical characteristics. If the patient presents with motor neuropathy, regardless of the degree of risk, rehabilitation exercises should be performed to limit the progression of the deformities. In those with mild to moderate PAD (IWGDF risk 1, 2, or 3), the healthcare provider should consider adding specific exercises to improve vascular function.

Several publications detail the key footwear aspects to consider.^47^, ^48^, ^49^ Others support the use of tilting soles and custom-made insoles.^48^^,^^50^ However, there is limited evidence about what type of shoes and socks should be recommended, that takes into account the condition of the patient's foot and their medical history. Our Delphi study addresses this information gap, compiling expert advice on appropriate socks and footwear during physical activity, according to the foot risk, taking into account possible complications like neuropathy, foot deformities, and PAD. For example, the type of sock should be tailored to the physical activity performed, but all cases should be light-colored (to visualize any bleeding more easily), clean, seamless, and with no tight-fitting elastics. If the patient has PAD and or neuropathy, the socks should have no seams, rubber, or elastic that could restrict circulation. However, consensus was not possible on certain aspects, such as the type of sock most appropriate for patients. We obtained no specific recommendations regarding shoes, but it was agreed that "a professional should always be consulted about the type of footwear before the patient begins an exercise regimen”.

A question that remains to be clarified is that of the appropriateness of exercise by patients with an open ulcer. To date, no guidelines have specifically addressed when and how patients should return to physical activity after an ulceration, although some studies have made observations in this regard. For example, Lyu et al.,^51^ argue that the early adoption of walking exercise can improve outcomes in patients with PAD. However, the current evidence is scarce and heterogeneous, as reflected in two systematic reviews which conclude that the available data do not allow any recommendations to be made on exercise in patients with diabetic foot ulcers.^14^^,^^52^

Pending new studies that address this topic, in the presence of PAD, our study offers expert recommendations on mobilizing the foot when there is ulceration. Another current issue is when and how to start or resume exercise after a recent ulceration. Our panelists agreed that patients should gradually ease into their exercise program, a view that is consistent with the results of other studies, suggesting that a well-controlled activity regimen that includes walking, maintaining balance, and strengthening the legs can positively influence the patient's health and function.^27^

The recommendations for which we failed to reach consensus reflected a similar lack of agreement in the scientific literature, relating to aspects such as the correct way to cut nails, the best type of socks for patients with neuropathy and PAD, and the use of smart watches or mobile apps to monitor blood oxygen (due to the absence of available devices with sufficiently reliable measurements). Given the dearth of quality studies or evidence on clinical efficacy, the panelists also failed to reach a consensus on recommendations for exercise in patients with IWGDF risk 2 or 3, the use of static pedals, the best type of surface on which to practice physical activity, or the use of a definitive offloading orthosis within 15 days of ulceration.

Some of these recommendations for which no consensus was reached, such as the type of sock or the correct cut for nails, are specific to the field of podiatry, while others (for example, the frequency and intensity of mobility exercises, the use of static pedals by patients at high risk of ulceration) correspond more to the field of sports medicine. Nevertheless, in our Delphi study, the relevant specialties were well distributed among the panelists. The lack of consensus regarding the more specific items might be due to the neutral position adopted by the experts who lacked specialist knowledge in these areas, a factor that may have introduced some bias into the study. To counter this possibility, we believe that recommendations on specific aspects of the foot or on types and repetitions of exercises should be agreed upon by a panel composed of experts within a single discipline.

The limitations to our research are those inherent to this type of qualitative study, such as the constraints of a close-ended survey, although free text space was provided for each item for panelists to freely comment. Another limitation, is the convenience nature of the sampling procedure used and the voluntary participation of the experts. Either or both of these factors may have introduced a selection bias. The low response rate in the initial selection of experts may be due to the difficulty getting in touch with the experts, because contact information was retrieved from their scientific publications, which in some cases may have been outdated. Consequently if the invitation was never received, this too would have constituted a selection bias. Another limitation is the fact that patients were not included in the panel or consulted as part of the study. However, we intend to conduct a follow-up study in which the recommendations obtained will be shared and managed by a group of people with diabetes, to better adapt the recommendations and the vocabulary to their needs.

Among the study's main strengths, to our knowledge this is the first set of expert recommendations to be proposed that address, in a clear and structured way, how patients should exercise, in accordance with their individual foot risk. Another strong point of this study is the high response rate obtained in each of the three rounds of the survey process (only one of the participating experts did not complete all three rounds). The Delphi methodology in itself represents another important strength, as its participatory nature favors the acceptability of the recommendations among multidisciplinary practitioners and their implementation in a range of clinical settings. On the other hand, these exercise recommendations have been formulated solely with the consensus of clinical experts; our research design method is not intended to be a replacement for robust, high-quality clinical trials, but rather a clinical guide to determining the patient's status and a starting point to consider important aspects for the individual patient. Further studies are needed to evaluate the ease of implementation of the recommendations, the degree of patient adherence, the long-term benefits, and the safety and efficacy of different types of physical exercise for this population.

## Conclusion

This Delphi study generated recommendations based on the consensus of international experts for physical activity and exercise by patient with diabetes at risk of ulceration. These recommendations take into account the state of the foot and the patient's history and status before physical activity. They include guidance such as when and how the foot should be examined, an initial consideration of the patient's general condition, the type of sock to be worn by those with PAD, neuropathy, and foot deformities, considering also the risk of foot ulcers (IWGDF risk 0–3), the type of exercise to be performed (in terms of intensity, duration, frequency, and progression), and recommendations for during and after physical activity/exercise, such as the use of custom-made plantar orthoses and shoe prescription, and the considerations for returning to physical activity after an ulceration.

## Conflicts of interest

The authors declare that they have no known competing financial interests or personal relationships that could have appeared to influence the work reported in this paper.

## Appendix Supplementary materials

Image, application 1

Image, application 2

## Footnotes

Supplementary material associated with this article can be found, in the online version, at doi:10.1016/j.bjpt.2023.100500.
