# Supplementary material for: Consensus-based recommendations on physical activity and exercise in patients with diabetes at risk of foot ulcerations: a Delphi study
Source: Braz J Phys Ther. 2023 Apr 6;27(2):100500. doi: 10.1016/j.bjpt.2023.100500 (PMC10201453; doi:10.1016/j.bjpt.2023.100500)
Supplement: Supplementary file 1 [file mmc1.pdf]

“Recommendations on physical activity and exercise in patients with diabetes and foot risk: Delphi consensus.” SURVEY 1ST ROUND OF DELPHI CONSENSUS

PARTICIPANT INFORMATION

We are contacting you to request your consent to participate in the research project titled: “Recommendations on physical activity and exercise in patients with diabetes and foot risk: Delphi consensus.”
This project has been approved by the Ethics Committee (Project Evaluation Body) Reference DCC.AGS.01.21 of the University. The project will be carried out in accordance with all the rules and ethical codes of conduct applicable to this research with human beings and the Code of Good Scientific Practices of the University.
Read the information provided and do not hesitate to ask us any questions that may arise. When you have understood the project, you will be asked to sign informed consent if you wish to participate in it.

If you decide to participate in this study you should know that you do so voluntarily and that you can also leave it at any time. In the event that you decide to suspend your participation, this will not entail any type of penalty or loss or damage to your rights and / or relationship with investigators.

INFORMATION FROM THE PRINCIPAL INVESTIGATOR OF THE PROJECT:
Name: main researcher
Contact email: main researcher
Contact phone: 6********

WHY IS THIS PROJECT BEING CARRIED OUT?
There are studies that show that physical exercise is beneficial for people with diabetes. But there are no known studies that demonstrate the possible adverse effects resulting from physical exercise on the foot of patients with diabetes and at-risk foot. In this study we intend to agree on the physical exercise guidelines to be recommended according to the risk of the patient's foot.

WHAT IS THE OBJECTIVE OF THE PROJECT?

Identify the physical exercise guidelines to be recommended according to the risk of the foot of the patient with diabetes through a consensus of experts.
The aim of establishing a consensus on exercise recommendations according to foot risk in people with diabetes.

HOW WILL THE STUDY BE CONDUCTED?

It is a consensus study conducted through the Delphi method.
The duration of the study will be extended for a period of time of 2 months, but this period may be longer or shorter (depending on the study).

WHAT BENEFITS CAN I GET FROM PARTICIPATING IN THIS STUDY?

You will receive the same treatment to participate or not in the project. As a result, you will not gain any direct benefit from your participation. However, the information you provide us, as well as that obtained from the analyses that are carried out, can be very useful to improve the knowledge we have today about physical exercise in patients with diabetes at risk and this will allow us to devise better forms of prevention, management and treatment than those we currently have.
For your participation in the study you will not get financial compensation.

WHAT RISKS CAN I SUFFER FROM PARTICIPATING IN THE STUDY?
none.

WHAT DATA WILL BE COLLECTED?
Name and surname.

HOW WILL MY PERSONAL DATA BE PROCESSED AND HOW WILL CONFIDENTIALITY BE PRESERVED?
The UMH, as Responsible for the processing of your personal data, informs you that these data will be treated in accordance with the provisions of Regulation (EU) 2016/679 of April 27 (RGPD) and Organic Law 3/2018 of December 5 (LOPDGDD)
Access to your personal information will be restricted to the principal investigator, when necessary, to verify the data and procedures of the study, but always maintaining the confidentiality of the same in accordance with current legislation. The Researcher, when processing and processing your data will take the appropriate measures to protect them and prevent access to them by unauthorized third parties.

* Responsible for the treatment: University.
* Internal data controller: main researcher
* Contact: In addition to being able to contact the principal investigator, you can contact the UMH data protection delegate: email data protection delegate
* Purpose: To carry out the processing of your personal data in order to participate in this research project
* Legitimation: Articles 6.1.a) of the RGPD: The interested party gives his explicit consent for the processing of his personal data for the realization of this research project.
* Obligation or not to provide data and consequences of not doing so: Not providing the requested data makes it impossible to comply with the purpose or purposes of the treatment.
* Automated decisions, profiles and applied logic: The data will not be used for automated decisions or for profiling.
* Recipients: There are no assignments to third parties
* International data transfer: Does not exist
* Conservation of data: They will be kept between 1 and 2 years to fulfill the purpose for which they were collected and to determine the possible responsibilities that may arise from this purpose and the processing of the data. Once the necessary time has elapsed, all the data will be deleted.

* Rights: The interested party may exercise their rights of access, rectification, opposition, deletion, portability and limitation of the treatment, as well as, not to be the object of decisions based solely on the automated processing of their data, for this they must be addressed by means of a request addressed to the attention of the General Secretary of the University. For any further consideration you can contact the data protection officer: Likewise, the interested party has the right to file a complaint with the Supervisory Authority if it considers that the treatment does not comply with current regulations
* Origin of the data: Own interested party
* Category of data of the interested parties: Those specified in the section: What data will be collected?
* Observations: To guarantee confidentiality, your data will be pseudonymized. This usually involves assigning a pseudonym to your data so that it can be processed without directly identifying you

WHO CAN I CONTACT WHEN IN DOUBT?
If you need more information about the study you can contact main researcher Phone: 6********; Email: main researcher.

*Obligatorio

INFORMED CONSENT TO PARTICIPATE IN THE STUDY

I have read this fact sheet and have had enough time to consider my decision.
I have been given the opportunity to ask questions and all of them have been satisfactorily answered.

I understand that my participation is voluntary.

I understand that I can withdraw from the study:
whenever
Without having to give explanations.
Having pondered the information I have been provided with, I declare that my decision is as follows:

Do you consent to participation in this research project? In addition, do you take for granted the use of your personal data under the conditions detailed in the information sheet? *

Marca solo un óvalo.

- Yes. I agree to participate in this study.
- No

Indicate your given name(s) and surname(s). We remind you that your participation is totally anonymous for the rest of the panelists, so your identity will only be known to the research team. *

What is your medical specialty? (endocrinologist, podiatrist, vascular surgeon, physical therapist, sports medicine specialist...) *

Recommendations prior to survey completion:

This is the first of three survey rounds; it has 50 items, consisting of different statements. Please indicate your level of agreement or disagreement on a scale of 1 to 5, where:
1 = indicates that you strongly disagree with the statement.
2 = indicates that you disagree.
3 = indicates that you neither agree nor disagree.
4 = indicates that you agree.
5 = indicates that you strongly agree.

The expert panel is an international, multidisciplinary group of specialists, including endocrinologists, podiatrists, physical therapists, and sports medicine specialists.
If you are unsure of your response or it relates to an area outside of your specialty, please mark number ‘3’ (neither agree nor disagree) At the end of every question, there is also space for commenting aspects that have not been covered or qualifying your response.
We remind you that this is the longest survey of the three rounds, as subsequent rounds will exclude items for which a consensus exists. Please allot approximately 20 minutes to complete the survey, for which you need only to indicate your level of agreement or disagreement with the statements proposed.

The research team thanks you sincerely for your valuable participation.

Cordially,
main researcher and the research team

General recommendations prior to commencing physical activity.

General recommendations for people at any IWGDF risk (categories 0–3) prior to commencing physical activity.

1. The patient themselves should inspect both feet before beginning physical activity, checking for:

1.1 Moisturization of the feet, assessing potential dryness of the forefoot, heels, or other areas. *

Marca solo un óvalo.

	1	2	3	4	5	
Strongly disagree.	◯	◯	◯	◯	◯	Strongly agree.

1.2 Appropriateness of the toenail cut. *

Marca solo un óvalo.

	1	2	3	4	5	
Strongly disagree.						Strongly agree.

1.3 Adequate length of the toenails. *

Marca solo un óvalo.

	1	2	3	4	5	
Strongly disagree.						Strongly agree.

1.4 Sharp edges on toenails. *

	1	2	3	4	5	
Strongly disagree.						Strongly agree.

1.5 Presence of hyperkeratosis or calluses. *

Marca solo un óvalo.

	1	2	3	4	5	
Strongly disagree.						Strongly agree.

1.6 Presence of blisters. *

Marca solo un óvalo.

	1	2	3	4	5	
Strongly disagree.	◯	◯	◯	◯	◯	Strongly agree.

1.7 Presence of wounds. *

Marca solo un óvalo.

	1	2	3	4	5	
Strongly disagree.	◯	◯	◯	◯	◯	Strongly agree.

1.8 Presence of irregularities, wear and tear, or other alterations on the inside of footwear. *

Marca solo un óvalo.

	1	2	3	4	5	
Strongly disagree.	◯	◯	◯	◯	◯	Strongly agree.

*Please mention any aspect related to this item that is not covered here (optional):

____

____

____

____

____

2. An initial examination of the patient’s general condition should be undertaken, including an assessment of:

2.1 Body mass index (BMI). *

Marca solo un óvalo.

	1	2	3	4	5	
Strongly disagree.						Strongly agree.

2.2 Age. *

Marca solo un óvalo.

	1	2	3	4	5	
Strongly disagree.						Strongly agree.

2.3 Possible cardiovascular alterations. *

Marca solo un óvalo.

	1	2	3	4	5	
Strongly disagree.						Strongly agree.

2.4 Evaluation of limited joint mobility. *

Marca solo un óvalo.

	1	2	3	4	5	
Strongly disagree.						Strongly agree.

2.5 Existence of arterial hypertension. *

Marca solo un óvalo.

	1	2	3	4	5	
Strongly disagree.						Strongly agree.

2.6 Uncontrolled retinopathy. *

Marca solo un óvalo.

	1	2	3	4	5	
Strongly disagree.						Strongly agree.

*Please mention any aspect related to this item that is not covered here (optional):

3. With regard to the diabetes, the attending physician or healthcare provider should assess the type (1 or 2), the level of metabolic control, and the treatment in order to determine the risk of hypoglycemia during physical activity. *

Marca solo un óvalo.

	1	2	3	4	5	
Strongly disagree.						Strongly agree.

*Please mention any aspect related to this item that is not covered here (optional):

4. People under treatment with insulin and/or sulfonylureas:

4.1 Should take a glucose reading before exercising. *

Marca solo un óvalo.

	1	2	3	4	5	
Strongly disagree.						Strongly agree.

4.2 Should take a glucose reading every 60 min during exercise. *

Marca solo un óvalo.

	1	2	3	4	5	
Strongly disagree.						Strongly agree.

4.3 Should take a glucose reading after exercising. *

Marca solo un óvalo.

	1	2	3	4	5	
Strongly disagree.						Strongly agree.

4.4 Should make certain modifications to their diet and/or pharmacological treatment. *

Marca solo un óvalo.

	1	2	3	4	5	
Strongly disagree.	◯	◯	◯	◯	◯	Strongly agree.

*Please mention any aspect related to this item that is not covered here (optional):

5. The presence of keratotic lesions or excessive dryness on different areas of the foot:

5.1 Should condition the intensity and duration of the exercise regimen recommended to the patient. *

Marca solo un óvalo.

	1	2	3	4	5	
Strongly disagree.	◯	◯	◯	◯	◯	Strongly agree.

5.2 Should condition the type of exercise recommended. *

Marca solo un óvalo.

	1	2	3	4	5	
Strongly disagree.	◯	◯	◯	◯	◯	Strongly agree.

*Please mention any aspect related to this item that is not covered here (optional):

6. The type of sock recommended should be conditioned by:

6.1 The presence of complications like neuropathy, foot deformities, or peripheral artery disease (PAD). *

Marca solo un óvalo.

	1	2	3	4	5	
Strongly disagree.						Strongly agree.

6.2 The type of physical activity recommended. *

Marca solo un óvalo.

	1	2	3	4	5	
Strongly disagree.						Strongly agree.

*Please mention any aspect related to this item that is not covered here (optional):

7. In the case of PAD (peripheral artery disease), the patient should use:

7.1 Socks made of natural fibers that help regulate foot temperature. *

Marca solo un óvalo.

	1	2	3	4	5	
Strongly disagree.						Strongly agree.

7.2 Socks without seams, rubber, or elastic that could compromise circulation. *

Marca solo un óvalo.

	1	2	3	4	5	
Strongly disagree.						Strongly agree.

7.3 Socks made with a blend of synthetic fibers. *

Marca solo un óvalo.

	1	2	3	4	5	
Strongly disagree.						Strongly agree.

7.4 Continuous temperature-monitoring socks. *

Marca solo un óvalo.

	1	2	3	4	5	
Strongly disagree.						Strongly agree.

7.5 Socks with antimicrobial compounds. *

Marca solo un óvalo.

	1	2	3	4	5	
Strongly disagree.	⬭	⬭	⬭	⬭	⬭	Strongly agree.

*Please mention any aspect related to this item that is not covered here (optional):

8. In case of neuropathy, patients should use:

8.1 Socks made of natural fibers. *

Marca solo un óvalo.

	1	2	3	4	5	
Strongly disagree.	⬭	⬭	⬭	⬭	⬭	Strongly agree.

8.2 Socks made with a blend of synthetic fibers that wick moisture away from the foot. *

Marca solo un óvalo.

	1	2	3	4	5	
Strongly disagree.	⬭	⬭	⬭	⬭	⬭	Strongly agree.

8.3 Socks without seams, rubber, or elastic. *

Marca solo un óvalo.

	1	2	3	4	5	
Strongly disagree.	◯	◯	◯	◯	◯	Strongly agree.

8.4 Continuous temperature-monitoring socks. *

Marca solo un óvalo.

	1	2	3	4	5	
Strongly disagree.	◯	◯	◯	◯	◯	Strongly agree.

8.5 Socks with antimicrobial compounds. *

Marca solo un óvalo.

	1	2	3	4	5	
Strongly disagree.	◯	◯	◯	◯	◯	Strongly agree.

*Please mention any aspect related to this item that is not covered here (optional):

9. Regarding the type of footwear, the healthcare provider should always be consulted before beginning the exercise regimen. *

Marca solo un óvalo.

	1	2	3	4	5	
Strongly disagree.	◯	◯	◯	◯	◯	Strongly agree.

*Please mention any aspect related to this item that is not covered here (optional):

10. The type of exercise recommended should take into account the patient’s preferences. *

Marca solo un óvalo.

	1	2	3	4	5	
Strongly disagree.	◯	◯	◯	◯	◯	Strongly agree.

*Please mention any aspect related to this item that is not covered here (optional):

11. The healthcare provider should recommend taking part in group exercise activities with people that share similar characteristics with the patient. *

Marca solo un óvalo.

	1	2	3	4	5	
Strongly disagree.	◯	◯	◯	◯	◯	Strongly agree.

*Please mention any aspect related to this item that is not covered here (optional):

____

____

____

____

____

12. The use of a smart watch or mobile apps during exercise is advised in patients with high cardiovascular risk in order to monitor:

12.1 Pulse. *

Marca solo un óvalo.

	1	2	3	4	5	
Strongly disagree.	◯	◯	◯	◯	◯	Strongly agree.

12.2 Blood pressure. *

Marca solo un óvalo.

	1	2	3	4	5	
Strongly disagree.	◯	◯	◯	◯	◯	Strongly agree.

12.3 Intensity of physical activity *

Marca solo un óvalo.

	1	2	3	4	5	
Strongly disagree.	◯	◯	◯	◯	◯	Strongly agree.

12.4 Type and duration of physical activity. *

Marca solo un óvalo.

	1	2	3	4	5	
Strongly disagree.	◯	◯	◯	◯	◯	Strongly agree.

12.5 Blood oxygen level, with accredited pulse oximeter. *

Marca solo un óvalo.

	1	2	3	4	5	
Strongly disagree.	◯	◯	◯	◯	◯	Strongly agree.

*Please mention any aspect related to this item that is not covered here (optional):

____
____
____
____
____

13. Patients at risk of hypoglycemia should:

13.1 Take fast-acting carbohydrates with them during exercise sessions. *

Marca solo un óvalo.

	1	2	3	4	5	
Strongly disagree.	◯	◯	◯	◯	◯	Strongly agree.

13.2 Take slow-release carbohydrates with them during the exercise session. *

Marca solo un óvalo.

	1	2	3	4	5	
Strongly disagree.	◯	◯	◯	◯	◯	Strongly agree.

13.3 Drink liquids during exercise. *

Marca solo un óvalo.

	1	2	3	4	5	
Strongly disagree.	◯	◯	◯	◯	◯	Strongly agree.

*Please mention any aspect related to this item that is not covered here (optional):

14. Regarding the characteristics of the exercise, it should be progressive, with moderate intensity in the first sessions and gradually becoming more vigorous according to the patient’s circumstances and ability. *

Marca solo un óvalo.

	1	2	3	4	5	
Strongly disagree.	⬭	⬭	⬭	⬭	⬭	Strongly agree.

*Please mention any aspect related to this item that is not covered here (optional):

15. In case of water activities, patients should use preventive measures to avoid foot infections. *

Marca solo un óvalo.

	1	2	3	4	5	
Strongly disagree.	⬭	⬭	⬭	⬭	⬭	Strongly agree.

*Please mention any aspect related to this item that is not covered here (optional):

Specific recommendations prior to physical activity for people with IWGDF risk 1, 2, and 3:

16. In patients with IWGDF risk 1, 2, or 3, the presence of any keratotic lesions or blisters should preclude any physical activity pending consultation with a healthcare professional. *

Marca solo un óvalo.

	1	2	3	4	5	
Strongly disagree.	⬭	⬭	⬭	⬭	⬭	Strongly agree.

*Please mention any aspect related to this item that is not covered here (optional):

____

____

____

____

____

17. Patients with hyperkeratosis, prior amputations, or calluses on the sole (IWGDF 2 or 3) should wear therapeutic footwear, including custom-made shoes, and receive orthopedic/podiatric treatment to redistribute areas of hyperpressure before doing any exercise. *

Marca solo un óvalo.

	1	2	3	4	5	
Strongly disagree.	⬭	⬭	⬭	⬭	⬭	Strongly agree.

*Please mention any aspect related to this item that is not covered here (optional):

18. Patients with IWGDF 2 or 3 should inspect their feet:

18.1 Before exercise. *

Marca solo un óvalo.

	1	2	3	4	5	
Strongly disagree.						Strongly agree.

18.2 During exercise. *

Marca solo un óvalo.

	1	2	3	4	5	
Strongly disagree.						Strongly agree.

18.3 On finishing exercise. *

Marca solo un óvalo.

	1	2	3	4	5	
Strongly disagree.						Strongly agree.

18.4 Foot inspections should consider temperature, color, and signs of lesions. *

Marca solo un óvalo.

	1	2	3	4	5	
Strongly disagree.	◯	◯	◯	◯	◯	Strongly agree.

18.5 Foot inspections should consider sensations described by the patient, such as pain, paresthesia (pins and needles), or itching. *

Marca solo un óvalo.

	1	2	3	4	5	
Strongly disagree.	◯	◯	◯	◯	◯	Strongly agree.

*Please mention any aspect related to this item that is not covered here (optional):

____

____

____

____

____

19. In case of neuropathy, patients should consult a podiatrist for:

19.1 A biomechanical study and orthopedic/podiatric treatment if appropriate. *

Marca solo un óvalo.

	1	2	3	4	5	
Strongly disagree.	◯	◯	◯	◯	◯	Strongly agree.

19.2 A prescription for biomechanical footwear with a flexible, semirigid sole at IWGDF 2 . *

Marca solo un óvalo.

	1	2	3	4	5	
Strongly disagree.	⬭	⬭	⬭	⬭	⬭	Strongly agree.

19.3 A recommendation on the use of biomechanical footwear with a rigid sole at IWGDF 3. *

Marca solo un óvalo.

	1	2	3	4	5	
Strongly disagree.	⬭	⬭	⬭	⬭	⬭	Strongly agree.

*Please mention any aspect related to this item that is not covered here (optional):

____

____

____

____

____

Specific recommendations prior to physical activity for people with IWGDF risk 3:

20. Patients who have had an active ulceration can begin putting weight on the foot 15 days after epithelialization is finished, without temporary offloading. *

Marca solo un óvalo.

	1	2	3	4	5	
Strongly disagree.	⬭	⬭	⬭	⬭	⬭	Strongly agree.

*Please mention any aspect related to this item that is not covered here (optional):

21. If the ulcer had a plantar location and has completely healed (the wound has not opened for 15 days after epithelialization), before beginning exercise, the patient should:

21.1 Receive personalized orthopedic/podiatric treatment. *

Marca solo un óvalo.

	1	2	3	4	5	
Strongly disagree.						Strongly agree.

21.2 Use biomechanical footwear with a rigid sole. *

Marca solo un óvalo.

	1	2	3	4	5	
Strongly disagree.						Strongly agree.

21.3 In case of amputation, the stump should be checked to ensure an even distribution in the pressure zones before supporting a load. *

Marca solo un óvalo.

	1	2	3	4	5	
Strongly disagree.						Strongly agree.

*Please mention any aspect related to this item that is not covered here (optional):

Type of exercise: intensity, duration, frequency, progression.

General recommendations for people at any IWGDF risk (categories 0–3) regarding the type of exercise: intensity, duration, frequency, progression.

22. When the patient spends a long period of time in a sedentary state, for every 30 min, they should spend at least 3 min walking around or stretching their legs and arms, regardless of whether they are regularly exercising. *

Marca solo un óvalo.

	1	2	3	4	5	
Strongly disagree.	⬭	⬭	⬭	⬭	⬭	Strongly agree.

*Please mention any aspect related to this item that is not covered here (optional):

23. Patients should be encouraged to perform aerobic activities as well as strength training and stretching, adapted to each type of at-risk foot. *

Marca solo un óvalo.

	1	2	3	4	5	
Strongly disagree.	⬭	⬭	⬭	⬭	⬭	Strongly agree.

*Please mention any aspect related to this item that is not covered here (optional):

24. In patients receiving rehabilitation treatment, telemonitoring of physical activity can improve adherence to treatment in patients with all categories of foot risk. *

Marca solo un óvalo.

	1	2	3	4	5	
Strongly disagree.	⬭	⬭	⬭	⬭	⬭	Strongly agree.

*Please mention any aspect related to this item that is not covered here (optional):

25. In people with diabetes, telemonitoring of physical activity can improve adherence to treatment in patients with all categories of foot risk. *

Marca solo un óvalo.

	1	2	3	4	5	
Strongly disagree.	◯	◯	◯	◯	◯	Strongly agree.

*Please mention any aspect related to this item that is not covered here (optional):

26. All programmed sessions of physical activity should include:

26.1 A warm-up prior to exercise of at least 5 min. *

Marca solo un óvalo.

	1	2	3	4	5	
Strongly disagree.	◯	◯	◯	◯	◯	Strongly agree.

26.2 Gentle stretching prior to commencing the exercise. *

Marca solo un óvalo.

	1	2	3	4	5	
Strongly disagree.	◯	◯	◯	◯	◯	Strongly agree.

26.3 On finishing the exercise, a cool-down of at least 5 min of slow walking. *

Marca solo un óvalo.

	1	2	3	4	5	
Strongly disagree.	◯	◯	◯	◯	◯	Strongly agree.

26.4 On finishing the exercise, stretches in at least the muscles worked, even if the patient does not do any general stretching. *

Marca solo un óvalo.

	1	2	3	4	5	
Strongly disagree.	◯	◯	◯	◯	◯	Strongly agree.

*Please mention any aspect related to this item that is not covered here (optional):

27. Patients with neuropathy (IWGDF 1, 2 and 3) should:

27.1 Do exercises to improve static balance: one-legged poses, adapted to the patient’s individual characteristics and exercise programs, and performed in tandem on 2 or 3 non-consecutive days a week. *

Marca solo un óvalo.

	1	2	3	4	5	
Strongly disagree.	◯	◯	◯	◯	◯	Strongly agree.

27.2 Do exercises to improve dynamic balance: walking several meters in tandem, laterally, and backwards, on 2 or 3 non-consecutive days a week, with activities adapted to the patient’s individual characteristics and exercise programs. *

Marca solo un óvalo.

	1	2	3	4	5	
Strongly disagree.	◯	◯	◯	◯	◯	Strongly agree.

27.3 Begin with low- or moderate-intensity aerobic exercise, appropriate to the patient’s age and physical characteristics. *

Marca solo un óvalo.

	1	2	3	4	5	
Strongly disagree.	◯	◯	◯	◯	◯	Strongly agree.

*Please mention any aspect related to this item that is not covered here (optional):

28. In patients with neuropathy, rehabilitation exercises are advisable to improve or limit the progression of the neuropathy, regardless of IWGDF risk. *

Marca solo un óvalo.

	1	2	3	4	5	
Strongly disagree.	⬭	⬭	⬭	⬭	⬭	Strongly agree.

*Please mention any aspect related to this item that is not covered here (optional):

29. In patients with mild to moderate PAD (IWGDF 1, 2, or 3), the healthcare provider should consider adding specific exercises to improve vascular function *

Marca solo un óvalo.

	1	2	3	4	5	
Strongly disagree.	⬭	⬭	⬭	⬭	⬭	Strongly agree.

*Please mention any aspect related to this item that is not covered here (optional):

Specific recommendations for patients with IWGDF 0, according to the type of exercise: intensity, duration, frequency, progression.

30. The patient should be encouraged to do as much physical activity as possible (walking up the stairs instead of using the elevator, walking to shops instead of driving, etc.). *

Marca solo un óvalo.

	1	2	3	4	5	
Strongly disagree.	⬭	⬭	⬭	⬭	⬭	Strongly agree.

*Please mention any aspect related to this item that is not covered here (optional):

____

____

____

____

____

31. In the absence of any cardiovascular alterations, the patient should:

31.1 Do aerobic activity. *

Marca solo un óvalo.

	1	2	3	4	5	
Strongly disagree.	⬭	⬭	⬭	⬭	⬭	Strongly agree.

31.2 Gradually increase daily moderate aerobic activity from 30 min/day to 1 h/day. *

Marca solo un óvalo.

	1	2	3	4	5	
Strongly disagree.	◯	◯	◯	◯	◯	Strongly agree.

.

31.3 Do aerobic exercise at least 3 days/week, without going 2 consecutive days without exercise. *

Marca solo un óvalo.

	1	2	3	4	5	
Strongly disagree.	◯	◯	◯	◯	◯	Strongly agree.

*Please mention any aspect related to this item that is not covered here (optional):

32. Exercise activities should include strength training: from passive movements of the ankle joint to active resistance (using a band) to work the ankle (dorsi and plantar flexion), the forefoot (inversion-eversion) and the toes (flexion-extension, abduction-adduction) at least twice a week. *

Marca solo un óvalo.

	1	2	3	4	5	
Strongly disagree.	◯	◯	◯	◯	◯	Strongly agree.

*Please mention any aspect related to this item that is not covered here (optional):

Specific recommendations for patients with IWGDF 1. According to the type of exercise: intensity, duration, frequency, progression.

33. Patients with peripheral neuropathy should:

33.1 Walk (moderate exercise) for 1 h/3 times a week, beginning with 30 min sessions. *

Marca solo un óvalo.

	1	2	3	4	5	
Strongly disagree.						Strongly agree.

33.2 Do strength training: resisted active movements (using a band), working the ankle (dorsi and plantar flexion), the forefoot (inversion-eversion) and the toes (flexion-extension, abduction-adduction) at least twice a week. *

Marca solo un óvalo.

	1	2	3	4	5	
Strongly disagree.						Strongly agree.

33.3 In case of coexisting obesity, do aerobic activity that does not overload joints or put excessive pressure on the feet (cycling, swimming). *

Marca solo un óvalo.

	1	2	3	4	5	
Strongly disagree.	◯	◯	◯	◯	◯	Strongly agree.

33.4 In case of cycling, use an offloading device on the pedal to distribute plantar stress if the patient has a specific area of hyperkeratosis. *

Marca solo un óvalo.

	1	2	3	4	5	
Strongly disagree.	◯	◯	◯	◯	◯	Strongly agree.

*Please mention any aspect related to this item that is not covered here (optional):

____

____

____

____

____

34. Patients with mild to moderate PAD (Rutherford grade 1 or 2) without neuropathy should begin walking at a moderate intensity and gradually increase it based on the onset of pain due to intermittent claudication. *

Marca solo un óvalo.

	1	2	3	4	5	
Strongly disagree.	◯	◯	◯	◯	◯	Strongly agree.

*Please mention any aspect related to this item that is not covered here (optional):

Specific recommendations for patients with IWGDF 2, according to the type of exercise: intensity, duration, frequency, progression.

35. Patients with IWGDF 2, neuropathy and foot deformity should:

35.1 Do low-intensity aerobic exercise, walking for 15-20 min 3 times a week or every other day. *

Marca solo un óvalo.

	1	2	3	4	5	
Strongly disagree.	⬭	⬭	⬭	⬭	⬭	Strongly agree.

35.2 In case of cycling, use an offloading device on the pedal to distribute plantar stress if the patient has a specific area of hyperkeratosis. *

Marca solo un óvalo.

	1	2	3	4	5	
Strongly disagree.	⬭	⬭	⬭	⬭	⬭	Strongly agree.

35.3 Add range-of-motion exercises: passive movements to the extent possible in the ankle joints (dorsi and plantar flexion), the forefoot (inversion-eversion) and the toes (flexion-extension, abduction-adduction) at least twice a week. *

Marca solo un óvalo.

	1	2	3	4	5	
Strongly disagree.	⬭	⬭	⬭	⬭	⬭	Strongly agree.

*Please mention any aspect related to this item that is not covered here (optional):

36. Patients with IWGDF 2, neuropathy and foot deformity should go swimming or participate in other aquatic activities that do not put pressure on the foot, 2 or 3 times a week. *

Marca solo un óvalo.

	1	2	3	4	5	
Strongly disagree.	⬭	⬭	⬭	⬭	⬭	Strongly agree.

*Please mention any aspect related to this item that is not covered here (optional):

37. Patients with IWGDF 2, neuropathy and foot deformity should use a stationary pedal exerciser in a seated, strain-free position for 15 min to 20 min a day, at least 4 days a week, without going 2 consecutive days without physical exercise. *

Marca solo un óvalo.

	1	2	3	4	5	
Strongly disagree.	⬭	⬭	⬭	⬭	⬭	Strongly agree.

*Please mention any aspect related to this item that is not covered here (optional):

Specific recommendations for patients with IWGDF 3, according to the type of exercise: intensity, duration, frequency, progression.

38. The healthcare provider should assess the patient’s routine physical activity, eliminating or modifying some activities if the patient presents lengthy, unregulated walking or physical efforts in excess of their level of tolerance. *

Marca solo un óvalo.

	1	2	3	4	5	
Strongly disagree.	◯	◯	◯	◯	◯	Strongly agree.

*Please mention any aspect related to this item that is not covered here (optional):

39. Patients with open ulcer should not put any pressure on the lesion or perform any exercises that put weight on the area where the ulcer is located. *

Marca solo un óvalo.

	1	2	3	4	5	
Strongly disagree.	◯	◯	◯	◯	◯	Strongly agree.

*Please mention any aspect related to this item that is not covered here (optional):

40. Patients with IWGDF 3 and an open ulcer without PAD should:

40.1 Do stretching exercises and strength training in a seated or supine position, with all activities adapted to the patient’s individual characteristics. *

Marca solo un óvalo.

	1	2	3	4	5	
Strongly disagree.	◯	◯	◯	◯	◯	Strongly agree.

40.2 Work on ankle mobility, plantar flexion, dorsiflexion, inversion, eversion, circumduction, and dorsi and plantar flexion of the toes at least 3 times a week or every other day, adapting all activities to the patient’s characteristics. *

Marca solo un óvalo.

	1	2	3	4	5	
Strongly disagree.	◯	◯	◯	◯	◯	Strongly agree.

40.3 In each set of mobility exercises, do at least 5 to 10 exercises with 10 to 15 repetitions each, 3 times a week or every other day, adapting all activities to the patient’s characteristics. *

Marca solo un óvalo.

	1	2	3	4	5	
Strongly disagree.	◯	◯	◯	◯	◯	Strongly agree.

*Please mention any aspect related to this item that is not covered here (optional):

41. Patients with severe PAD (with lesions) should:

41.1 Do mobility exercises of the lower limbs in a seated or supine position. *

Marca solo un óvalo.

	1	2	3	4	5	
Strongly disagree.						Strongly agree.

41.2 Work on ankle mobility, plantar flexion, dorsiflexion, inversion, eversion, circumduction, and dorsi and plantar flexion of the toes at least 3 times a week or every other day. *

Marca solo un óvalo.

	1	2	3	4	5	
Strongly disagree.						Strongly agree.

41.3 Adapt all exercise sets to the symptomology related to the lesion and its location. *

Marca solo un óvalo.

	1	2	3	4	5	
Strongly disagree.						Strongly agree.

*Please mention any aspect related to this item that is not covered here (optional):

42. Patients with IWGDF 3 and a recent (< 15 days) history of ulceration should:

42.1 Begin by doing at least 10 min of daily activity with a technical aid (cane, crutch) and gradually progress over the following 15 days if their condition allows it. *

Marca solo un óvalo.

	1	2	3	4	5	
Strongly disagree.	⬭	⬭	⬭	⬭	⬭	Strongly agree.

42.2 Begin by doing at least 10 min of daily activity and gradually progress over the following 15 days if their condition allows it. *

Marca solo un óvalo.

	1	2	3	4	5	
Strongly disagree.	⬭	⬭	⬭	⬭	⬭	Strongly agree.

*Please mention any aspect related to this item that is not covered here (optional):

43. At one month of healing, patients with IWGDF 3 should:

43.1 Begin walking 15 min to 20 min a day, 3 days a week, at a low intensity. *

Marca solo un óvalo.

	1	2	3	4	5	
Strongly disagree.	⬭	⬭	⬭	⬭	⬭	Strongly agree.

43.2 Go swimming or do aquatic activities that do not put pressure on the foot, 2 or 3 times a week. *

Marca solo un óvalo.

	1	2	3	4	5	
Strongly disagree.	⬭	⬭	⬭	⬭	⬭	Strongly agree.

43.3 Use a stationary pedal exerciser in a seated position, starting with 15 min to 20 min a day, 3 days a week or every other day. *

Marca solo un óvalo.

	1	2	3	4	5	
Strongly disagree.	⬭	⬭	⬭	⬭	⬭	Strongly agree.

43.4 Do yoga or other stretching and balance exercises, 2 to 3 times a week. *

Marca solo un óvalo.

	1	2	3	4	5	
Strongly disagree.						Strongly agree.

*Please mention any aspect related to this item that is not covered here (optional):

44. Patients with IWGDF 3 and a prior amputation should consult a rehabilitation specialist to strengthen the stump and treat the phantom limb with functional exercises. *

Marca solo un óvalo.

	1	2	3	4	5	
Strongly disagree.						Strongly agree.

*Please mention any aspect related to this item that is not covered here (optional):

Recommendations during exercise

Specific recommendations for patients with IWGDF 1, 2, or 3

45. Patients with an at-risk foot, neuropathy, and/or deformity should walk on shock-absorbing surfaces (grass, clay), without excluding other types of surfaces. *

Marca solo un óvalo.

	1	2	3	4	5	
Strongly disagree.	◯	◯	◯	◯	◯	Strongly agree.

*Please mention any aspect related to this item that is not covered here (optional):

46. The use of a cane should be considered in patients with neuropathy and foot deformity, in case of important alterations in proprioception. *

Marca solo un óvalo.

	1	2	3	4	5	
Strongly disagree.	◯	◯	◯	◯	◯	Strongly agree.

*Please mention any aspect related to this item that is not covered here (optional):

47. If the patient has a history of ulcers, they should use monitoring devices with alarms to control temperature and/or foot pressure during the activity. *

Marca solo un óvalo.

	1	2	3	4	5	
Strongly disagree.	◯	◯	◯	◯	◯	Strongly agree.

*Please mention any aspect related to this item that is not covered here (optional):

Recommendations following exercise

General recommendations following exercise for patients with all categories of foot risk.

48. Risk factors for ulceration in patients with all categories of IWGDF risk who are performing moderate- to high-intensity physical activity should be monitored at least as frequently as recommended by the IWGDF: IWGDF 0, once yearly; IWGDF 1, every 6 to 12 months; IWGDF 2, every 3 to 6 months; IWGDF 3, every 1 to 3 months. Examinations should also include a review of the footwear being used during physical activity exercises. *

Marca solo un óvalo.

	1	2	3	4	5	
Strongly disagree.						Strongly agree.

*Please mention any aspect related to this item that is not covered here (optional):

49. Healthcare providers should follow up patients with IWGDF 2 and 3 every month to monitor the recommended exercise regimen and modify it according to the patient’s evolution. *

Marca solo un óvalo.

	1	2	3	4	5	
Strongly disagree.						Strongly agree.

*Please mention any aspect related to this item that is not covered here (optional):

50. All patients with neuropathy who use custom-made soles should be followed at 15 days following their use. *

Marca solo un óvalo.

	1	2	3	4	5	
Strongly disagree.	◯	◯	◯	◯	◯	Strongly agree.

*Please mention any aspect related to this item that is not covered here (optional):

Este contenido no ha sido creado ni aprobado por Google.

Google Formularios
