# Supplementary material for: Consensus-based recommendations on physical activity and exercise in patients with diabetes at risk of foot ulcerations: a Delphi study
Source: Braz J Phys Ther. 2023 Apr 6;27(2):100500. doi: 10.1016/j.bjpt.2023.100500 (PMC10201453; doi:10.1016/j.bjpt.2023.100500)
Supplement: Supplementary file 2 [file mmc2.docx]

| **Supplementary material 2 – S.1.** Results of the 1ST round of the Delphi survey. |  |  |  |
| --- | --- | --- | --- |
| General recommendations prior to commencing physical activity.  General recommendations for people at any IWGDF risk (categories 0–3) prior to commencing physical activity. |  |  |  |
| **1. The patient themselves should inspect both feet before beginning physical activity, checking for:** | **Agree**  **(%)** | **Disagree**  **(%)** | **Neutral**  **(%)** |
| 1.1 Moisturization of the feet, assessing potential dryness of the forefoot, heels, or other areas. | 82.8 | 3.4 | 13.8 |
| 1.2 Appropriateness of the toenail cut. | 79.3 | 6.9 | 13.8 |
| 1.3 Adequate length of the toenails. | 89.7 | 3.4 | 6.9 |
| 1.4 Sharp edges on toenails. | 93.1 | 0 | 6.9 |
| 1.5 Presence of hyperkeratosis or calluses. | 93.1 | 0 | 6.9 |
| 1.6 Presence of blisters. | 96.6 | 0 | 3.4 |
| 1.7 Presence of wounds. | 100.0 | 0 | 0 |
| 1.8 Presence of irregularities, wear and tear, or other alterations on the inside of footwear. | 93.1 | 0 | 6.9 |
| **2. An initial examination of the patient’s general condition should be undertaken, including an assessment of:** | |  |  |
| 2.1 Body mass index (BMI). | 72.4 | 6.9 | 20.7 |
| 2.2 Age. | 82.8 | 3.4 | 13.8 |
| 2.3 Possible cardiovascular alterations. | 93.1 | 0 | 6.9 |
| 2.4 Evaluation of limited joint mobility. | 93.1 | 0 | 6.9 |
| 2.5 Existence of arterial hypertension. | 89.7 | 6.9 | 3.4 |
| 2.6 Uncontrolled retinopathy. | 79.3 | 0 | 20.7 |
| **3. With regard to the diabetes, the attending physician or healthcare provider should assess the type (1 or 2), the level of metabolic control, and the treatment in order to determine the risk of hypoglycemia during physical activity.** | 96.6 | 0 | 3.4 |
| **4. People under treatment with insulin and/or sulfonylureas:** | |  |  |
| 4.1 Should take a glucose reading before exercising. | 82.8 | 3.4 | 13.8 |
| 4.2 Should take a glucose reading every 60 min during exercise. | 65.5 | 3.4 | 31.0 |
| 4.3 Should take a glucose reading after exercising. | 82.8 | 3.4 | 13.8 |
| 4.4 Should make certain modifications to their diet and/or pharmacological treatment. | 72.4 | 3.4 | 24.1 |
| **5. The presence of keratotic lesions or excessive dryness on different areas of the foot:** | |  |  |
|  | **Agree**  **(%)** | **Disagree**  **(%)** | **Neutral**  **(%)** |
| 5.1 Should condition the intensity and duration of the exercise regimen recommended to the patient. | 69.0 | 17.2 | 13.8 |
| 5.2 Should condition the type of exercise recommended. | 79.3 | 13.8 | 6.9 |
| **6. The type of sock recommended should be conditioned by:** | |  |  |
| 6.1 The presence of complications like neuropathy, foot deformities, or peripheral artery disease (PAD). | 82.8 | 10.3 | 6.9 |
| 6.2 The type of physical activity recommended. | 65.5 | 20.7 | 13.8 |
| **7. In the case of PAD (peripheral artery disease), the patient should use:** | |  |  |
| 7.1 Socks made of natural fibers that help regulate foot temperature.v | 72.4 | 3.4 | 24.1 |
| 7.2 Socks without seams, rubber, or elastic that could compromise circulation. | 89.7 | 0 | 10.3 |
| 7.3 Socks made with a blend of synthetic fibers. | 13.8 | 31.0 | 55.2 |
| 7.4 Continuous temperature-monitoring socks. | 10.3 | 31.0 | 58.6 |
| 7.5 Socks with antimicrobial compounds. | 24.1 | 34.5 | 41.4 |
| **8. In case of neuropathy, patients should use:** | |  |  |
| 8. Socks made of natural fibers. | 62.1 | 3.4 | 34.5 |
| 8.2 Socks made with a blend of synthetic fibers that wick moisture away from the foot. | 27.6 | 20.7 | 51.7 |
| 8.3 Socks without seams, rubber, or elastic. | 89.7 | 0 | 10.3 |
| 8.4 Continuous temperature-monitoring socks. | 13.8 | 34.5 | 51.7 |
| 8.5 Socks with antimicrobial compounds. | 24.1 | 34.5 | 41.4 |
| **9. Regarding the type of footwear, the healthcare provider should always be consulted before beginning the exercise regimen.** | **79.3** | **3.4** | **17.2** |
| 10. The type of exercise recommended should take into account the patient’s preferences. | 93.1 | 0 | 6.9 |
| **11. The healthcare provider should recommend taking part in group exercise activities with people that share similar characteristics with the patient.** | 37.9 | 17.2 | 44.8 |
| **12. The use of a smart watch or mobile apps during exercise is advised in patients with high cardiovascular risk in order to monitor:** | |  |  |
| 12.1 Pulse. | 82.8 | 3.4 | 13.8 |
| 12.2 Blood pressure. | 72.4 | 10.3 | 17.2 |
|  | **Agree**  **(%)** | **Disagree**  **(%)** | **Neutral**  **(%)** |
| 12.3 Intensity of physical activity | 69.0 | 3.4 | 27.6 |
| 12.4 Type and duration of physical activity. | 72.4 | 6.9 | 20.7 |
| 12.5 Blood oxygen level, with accredited pulse oximeter. | 31.0 | 24.1 | 44.8 |
| **13. Patients at risk of hypoglycemia should:** | |  |  |
| 13.1 Take fast-acting carbohydrates with them during exercise sessions. | 93.1 | 0 | 6.9 |
| 13.2 Take slow-release carbohydrates with them during the exercise session. | 48.3 | 17.2 | 34.5 |
| 13.3 Drink liquids during exercise. | 79.3 | 3.4 | 17.2 |
| **14. Regarding the characteristics of the exercise, it should be progressive, with moderate intensity in the first sessions and gradually becoming more vigorous according to the patient’s circumstances and ability.** | 93.1 | 3.4 | 3.4 |
| **15. In case of water activities, patients should use preventive measures to avoid foot infections.** | 82.8 | 6.9 | 10.3 |
| **SPECIFIC RECOMMENDATIONS PRIOR TO PHYSICAL ACTIVITY FOR PEOPLE WITH IWGDF RISK 1, 2, AND 3:** | |  |  |
| **16. In patients with IWGDF risk 1, 2, or 3, the presence of any keratotic lesions or blisters should preclude any physical activity pending consultation with a healthcare professional.** | 82.8 | 13.8 | 3.4 |
| **17. Patients with hyperkeratosis, prior amputations, or calluses on the sole (IWGDF 2 or 3) should wear therapeutic footwear, including custom-made shoes, and receive orthopedic/podiatric treatment to redistribute areas of hyperpressure before doing any exercise.** | 96.6 | 0 | 3.4 |
| **18. Patients with IWGDF 2 or 3 should inspect their feet:** | |  |  |
| 18.1 Before exercise. | 100.0 | 0 | 0 |
| 18.2 During exercise. | 58.6 | 17.2 | 24.1 |
| 18.3 On finishing exercise. | 100.0 | 0 | 0 |
| 18.4 Foot inspections should consider temperature, color, and signs of lesions. | 96.6 | 0 | 3.4 |
| 18.5 Foot inspections should consider sensations described by the patient, such as pain, paresthesia (pins and needles), or itching. | 75.9 | 3.4 | 20.7 |
| **19. In case of neuropathy, patients should consult a podiatrist for:** | |  |  |
| 19.1 A biomechanical study and orthopedic/podiatric treatment if appropriate. | 100.0 | 0 | 0 |
| 19.2 A prescription for biomechanical footwear with a flexible, semirigid sole at IWGDF 2 . | 69.0 | 6.9 | 24.1 |
| 19.3 A recommendation on the use of biomechanical footwear with a rigid sole at IWGDF 3. | 65.5 | 3.4 | 31.0 |
|  | **Agree**  **(%)** | **Disagree**  **(%)** | **Neutral**  **(%)** |
| **SPECIFIC RECOMMENDATIONS PRIOR TO PHYSICAL ACTIVITY FOR PEOPLE WITH IWGDF RISK 3:** | |  |  |
| **20. Patients who have had an active ulceration can begin putting weight on the foot 15 days after epithelialization is finished, without temporary offloading.** | 27.6 | 44.8 | 27.6 |
| **21. If the ulcer had a plantar location and has completely healed (the wound has not opened for 15 days after epithelialization), before beginning exercise, the patient should:** | |  |  |
| 21.1 Receive personalized orthopedic/podiatric treatment. | 82.8 | 0 | 17.2 |
| 21.2 Use biomechanical footwear with a rigid sole. | 62.1 | 3.4 | 34.5 |
| 21.3 In case of amputation, the stump should be checked to ensure an even distribution in the pressure zones before supporting a load. | 96.6 | 0 | 3.4 |
| **TYPE OF EXERCISE: INTENSITY, DURATION, FREQUENCY, PROGRESSION.**  **General recommendations for people at any IWGDF risk (categories 0–3) regarding the type of exercise: intensity, duration, frequency, progression.** | |  |  |
| **22. When the patient spends a long period of time in a sedentary state, for every 30 min, they should spend at least 3 min walking around or stretching their legs and arms, regardless of whether they are regularly exercising.** | 62.1 | 3.4 | 34.5 |
| **23. Patients should be encouraged to perform aerobic activities as well as strength training and stretching, adapted to each type of at-risk foot.** | 93.1 | 0 | 6.9 |
| **24. In patients receiving rehabilitation treatment, telemonitoring of physical activity can improve adherence to treatment in patients with all categories of foot risk..** | 75.9 | 0 | 24.1 |
| **25. In people with diabetes, telemonitoring of physical activity can improve adherence to treatment in patients with all categories of foot risk.** | 72.4 | 3.4 | 24.1 |
| **26. All programmed sessions of physical activity should include:** | |  |  |
| 26.1 A warm-up prior to exercise of at least 5 min. | 89.7 | 3.4 | 6.9 |
| 26.2 Gentle stretching prior to commencing the exercise. | 82.8 | 6.9 | 10.3 |
| 26.3 On finishing the exercise, a cool-down of at least 5 min of slow walking. | 79.3 | 3.4 | 17.2 |
| 26.4 On finishing the exercise, stretches in at least the muscles worked, even if the patient does not do any general stretching. | 72.4 | 3.4 | 24.1 |
| **27. Patients with neuropathy (IWGDF 1, 2 and 3) should:** | **Agree**  **(%)** | **Disagree**  **(%)** | **Neutral**  **(%)** |
| 27.1 Do exercises to improve static balance: one-legged poses, adapted to the patient’s individual characteristics and exercise programs, and performed in tandem on 2 or 3 non-consecutive days a week. | 55.2 | 3.4 | 41.4 |
| 27.2 Do exercises to improve dynamic balance: walking several meters in tandem, laterally, and backwards, on 2 or 3 non-consecutive days a week, with activities adapted to the patient’s individual characteristics and exercise programs. | 62.1 | 6.9 | 31.0 |
| 27.3 Begin with low- or moderate-intensity aerobic exercise, appropriate to the patient’s age and physical characteristics. | 86.2 | 3.4 | 10.3 |
| **28. In patients with neuropathy, rehabilitation exercises are advisable to improve or limit the progression of the neuropathy, regardless of IWGDF risk. .** | 48.3 | 13.8 | 37.9 |
| **29. In patients with mild to moderate PAD (IWGDF 1, 2, or 3), the healthcare provider should consider adding specific exercises to improve vascular function** | 75.9 | 6.9 | 17.2 |
| **SPECIFIC RECOMMENDATIONS FOR PATIENTS WITH IWGDF 0, ACCORDING TO THE TYPE OF EXERCISE: INTENSITY, DURATION, FREQUENCY, PROGRESSION.** | |  |  |
| **30. The patient should be encouraged to do as much physical activity as possible (walking up the stairs instead of using the elevator, walking to shops instead of driving, etc.).** | 96.6 | 0 | 3.4 |
| **31. In the absence of any cardiovascular alterations, the patient should:** | |  |  |
| 31.1 Do aerobic activity. | 89.7 | 3.4 | 6.9 |
| 31.2 Gradually increase daily moderate aerobic activity from 30 min/day to 1 h/day. | 75.9 | 6.9 | 17.2 |
| 31.3 Do aerobic exercise at least 3 days/week, without going 2 consecutive days without exercise. | 65.5 | 3.4 | 31.0 |
| **32. Exercise activities should include strength training: from passive movements of the ankle joint to active resistance (using a band) to work the ankle (dorsi and plantar flexion), the forefoot (inversion-eversion) and the toes (flexion-extension, abduction-adduction) at least twice a week.** | 72.4 | 10.3 | 17.2 |
| **SPECIFIC RECOMMENDATIONS FOR PATIENTS WITH IWGDF 1. ACCORDING TO THE TYPE OF EXERCISE: INTENSITY, DURATION, FREQUENCY, PROGRESSION.** | |  |  |
| **33. Patients with peripheral neuropathy should:** | **Agree**  **(%)** | **Disagree**  **(%)** | **Neutral**  **(%)** |
| 33.1 Walk (moderate exercise) for 1 h/3 times a week, beginning with 30 min sessions. | 75.9 | 6.9 | 17.2 |
| 33.2 Do strength training: resisted active movements (using a band), working the ankle (dorsi and plantar flexion), the forefoot (inversion-eversion) and the toes (flexion-extension, abduction-adduction) at least twice a week. | 69.0 | 10.3 | 20.7 |
| 33.3 In case of coexisting obesity, do aerobic activity that does not overload joints or put excessive pressure on the feet (cycling, swimming). | 79.3 | 3.4 | 17.2 |
| 33.4 In case of cycling, use an offloading device on the pedal to distribute plantar stress if the patient has a specific area of hyperkeratosis. | 65.5 | 6.9 | 27.6 |
| **34. Patients with mild to moderate PAD (Rutherford grade 1 or 2) without neuropathy should begin walking at a moderate intensity and gradually increase it based on the onset of pain due to intermittent claudication.** | 79.3 | 3.4 | 17.2 |
| **SPECIFIC RECOMMENDATIONS FOR PATIENTS WITH IWGDF 2, ACCORDING TO THE TYPE OF EXERCISE: INTENSITY, DURATION, FREQUENCY, PROGRESSION.** | |  |  |
| **35. Patients with IWGDF 2, neuropathy and foot deformity should:** | |  |  |
| 35.1 Do low-intensity aerobic exercise, walking for 15-20 min 3 times a week or every other day. | 65.5 | 10.3 | 24.1 |
| 35.2 In case of cycling, use an offloading device on the pedal to distribute plantar stress if the patient has a specific area of hyperkeratosis. | 72.4 | 3.4 | 24.1 |
| 35.3 Add range-of-motion exercises: passive movements to the extent possible in the ankle joints (dorsi and plantar flexion), the forefoot (inversion-eversion) and the toes (flexion-extension, abduction-adduction) at least twice a week. | 72.4 | 3.4 | 24.1 |
| **36. Patients with IWGDF 2, neuropathy and foot deformity should go swimming or participate in other aquatic activities that do not put pressure on the foot, 2 or 3 times a week.** | 62.1 | 6.9 | 31.0 |
| 37. Patients with IWGDF 2, neuropathy and foot deformity should use a stationary pedal exerciser in a seated, strain-free position for 15 min to 20 min a day, at least 4 days a week, without going 2 consecutive days without physical exercise. | 44.8 | 6.9 | 48.3 |
| **SPECIFIC RECOMMENDATIONS FOR PATIENTS WITH IWGDF 3, ACCORDING TO THE TYPE OF EXERCISE: INTENSITY, DURATION, FREQUENCY, PROGRESSION.** | **Agree**  **(%)** | **Disagree**  **(%)** | **Neutral**  **(%)** |
| **38. The healthcare provider should assess the patient’s routine physical activity, eliminating or modifying some activities if the patient presents lengthy, unregulated walking or physical efforts in excess of their level of tolerance.** | 89.7 | 3.4 | 6.9 |
| **39. Patients with open ulcer should not put any pressure on the lesion or perform any exercises that put weight on the area where the ulcer is located.** | 93.1 | 0 | 6.9 |
| **40. Patients with IWGDF 3 and an open ulcer without PAD should:** |  |  |  |
| 40.1 Do stretching exercises and strength training in a seated or supine position, with all activities adapted to the patient’s individual characteristics. | 79.3 | 10.3 | 10.3 |
| 40.2 Work on ankle mobility, plantar flexion, dorsiflexion, inversion, eversion, circumduction, and dorsi and plantar flexion of the toes at least 3 times a week or every other day, adapting all activities to the patient’s characteristics. | 69.0 | 10.3 | 20.7 |
| 40.3 In each set of mobility exercises, do at least 5 to 10 exercises with 10 to 15 repetitions each, 3 times a week or every other day, adapting all activities to the patient’s characteristics. | 55.2 | 17.2 | 27.6 |
| **41. Patients with severe PAD (with lesions) should:** | |  |  |
| 41.1 Do mobility exercises of the lower limbs in a seated or supine position. | 86.2 | 10.3 | 3.4 |
| 41.2 Work on ankle mobility, plantar flexion, dorsiflexion, inversion, eversion, circumduction, and dorsi and plantar flexion of the toes at least 3 times a week or every other day. | 79.3 | 3.4 | 17.2 |
| 41.3 Adapt all exercise sets to the symptomology related to the lesion and its location. | 86.2 | 6.9 | 6.9 |
| **42. Patients with IWGDF 3 and a recent (< 15 days) history of ulceration should:** |  |  |  |
| 42.1 Begin by doing at least 10 min of daily activity with a technical aid (cane, crutch) and gradually progress over the following 15 days if their condition allows it. | 58.6 | 13.8 | 27.6 |
| 42.2 Begin by doing at least 10 min of daily activity and gradually progress over the following 15 days if their condition allows it. | 69.0 | 10.3 | 20.7 |
| **43. At one month of healing, patients with IWGDF 3 should:** | |  |  |
| 43.1 Begin walking 15 min to 20 min a day, 3 days a week, at a low intensity. | 58.6 | 3.4 | 37.9 |
| 43.2 Go swimming or do aquatic activities that do not put pressure on the foot, 2 or 3 times a week. | 58.6 | 6.9 | 34.5 |
| 43.3 Use a stationary pedal exerciser in a seated position, starting with 15 min to 20 min a day, 3 days a week or every other day. | 62.1 | 6.9 | 31.0 |
| 43.4 Do yoga or other stretching and balance exercises, 2 to 3 times a week. | 51.7 | 6.9 | 41.4 |
| **44. Patients with IWGDF 3 and a prior amputation should consult a rehabilitation specialist to strengthen the stump and treat the phantom limb with functional exercises.** | 86.2 | 3.4 | 10.3 |
| RECOMMENDATIONS DURING EXERCISE  Specific recommendations for patients with IWGDF 1, 2, or 3 | **Agree**  **(%)** | **Disagree**  **(%)** | **Neutral**  **(%)** |
| **45. Patients with an at-risk foot, neuropathy, and/or deformity should walk on shock-absorbing surfaces (grass, clay), without excluding other types of surfaces.** | 58.6 | 13.8 | 27.6 |
| **46. The use of a cane should be considered in patients with neuropathy and foot deformity, in case of important alterations in proprioception.** | 79.3 | 6.9 | 13.8 |
| **47. If the patient has a history of ulcers, they should use monitoring devices with alarms to control temperature and/or foot pressure during the activity.** | 48.3 | 20.7 | 31.0 |
| **RECOMMENDATIONS FOLLOWING EXERCISE**  **General recommendations following exercise for patients with all categories of foot risk.** | |  |  |
| **48. Risk factors for ulceration in patients with all categories of IWGDF risk who are performing moderate- to high-intensity physical activity should be monitored at least as frequently as recommended by the IWGDF: IWGDF 0, once yearly; IWGDF 1, every 6 to 12 months; IWGDF 2, every 3 to 6 months; IWGDF 3, every 1 to 3 months. Examinations should also include a review of the footwear being used during physical activity exercises.** | 86.2 | 0 | 13.8 |
| **49. Healthcare providers should follow up patients with IWGDF 2 and 3 every month to monitor the recommended exercise regimen and modify it according to the patient’s evolution.** | 79.3 | 3.4 | 17.2 |
| **50. All patients with neuropathy who use custom-made soles should be followed at 15 days following their use.** | 75.9 | 6.9 | 17.2 |

Bold type: main statement; Black table cell: Title thematic blocks; Grey table cell: Non-consensus items

| **Supplementary material 2 – S.2.** Changes after reviewing the panelists’ comments. | | |
| --- | --- | --- |
| **ITEMS 1ST round** | **Changes for 2ST round** | **Changes for 3ST round** |
| GENERAL RECOMMENDATIONS PRIOR TO COMMENCING PHYSICAL ACTIVITY. General recommendations for people at any IWGDF risk (categories 0–3) prior to commencing physical activity. | | |
| **1. The patient themselves should inspect both feet before beginning physical activity, checking for:** |  |  |
| 1.2 Appropriateness of the toenail cut trying a straight cut. | 1.2 Ensure proper toenail cutting, with a straight cut | 1.2. Cut toenails appropriately, according to their morphology. |
| **2. An initial examination of the patient’s general condition should be undertaken, including an assessment of:** |  |  |
| 2.1 Body mass index (BMI). To adapt the activity in cases of obesity. | 2.1 Body Mass Index (BMI). Recommended activity levels should take into account the presence of obesity. |  |
| 2.6 Uncontrolled retinopathy or nefropathy, that could be exercise type recomendations | 2.6 The presence of uncontrolled retinopathy or renal impairment may influence the type of exercise recommended. |  |
| **4. People under treatment with insulin and/or sulfonylureas:** |  |  |
| 4.2 Should take a glucose reading every 60 min during exercise. | 4.2 Glucose levels should be monitored during exercise, and more frequently for high-risk patients being treated with insulin. |  |
| 4.4 Should make certain modifications to their diet and/or pharmacological treatment. | 4.4 If necessary, dietary and/or pharmacological adjustments should be recommended by the healthcare provider, and a glycogen kit should be available. |  |
| **5. The presence of keratotic lesions or excessive dryness on different areas of the foot:** |  |  |
| 5.1 Should condition the intensity and duration of the exercise regimen recommended to the patient. | 5.1 These conditions should be treated before establishing the intensity and duration of the exercise recommended. |  |
| 5.2 Should condition the type of exercise recommended. | 5.2 These conditions should be treated before establishing the type of exercise recommended. |  |
| **6. The type of sock recommended should be conditioned by:** |  |  |
| 6.2 The type of physical activity recommended. | 6.2 Socks should suit the physical activity to be performed. They should be made of cotton, light coloured to make any bleeding easily visible, seamless and with no tight-fitting elastic. | 6.2. The type of sock to be recommended should be specific, according to the physical activity to be carried out, but preferably light-coloured to visualise any bleeding more easily, clean, seamless and with no tight-fitting elastics. |
| **7. In the case of PAD (peripheral artery disease), the patient should use:** |  |  |
| 7.1 Socks made of natural fibers that help regulate foot temperature. | 7.1 Socks made of natural fibers that help regulate foot temperature. | 7.1. Socks that help maintain the temperature of the foot should be recommended. |
| 7.3 Socks made with a blend of synthetic fibers | 7.3 Socks should contain a blend of synthetic fibres to wick moisture away from the foot. |  |
| 7.4 Continuous temperature-monitoring socks | 7.4 Socks should facilitate temperature monitoring in order to indicate possible injury during exercise. | 7.4. Socks providing continuous temperature control should be recommended, to reveal possible vascular injuries during exercise. |
| 7.5 Socks with antimicrobial compounds. | 7.5 Socks should incorporate antimicrobial compounds to reduce the risk of foot infections. | 7.5. Socks that incorporate antibacterial compounds should be recommended, to reduce the risk of infection in patients with vascular lesions. |
| **8. In case of NEUROPATHY, patients should use:** |  |  |
| 8.1 Socks made of natural fibers. | 8.1 Socks made of natural fibers that help regulate foot temperature. |  |
| 8.4 Continuous temperature-monitoring socks. | 8.4 Socks should facilitate temperature monitoring in order to indicate possible injury during exercise. |  |
| 8.5 Socks with antimicrobial compounds. | 8.5 Socks should incorporate antimicrobial compounds to reduce the risk of foot infections. |  |
| **9. Regarding the type of footwear, the healthcare provider should always be consulted before beginning the exercise regimen.** | 9. A professional should always be consulted about the type of footwear before the patient begins an exercise regimen.) |  |
| 11. The healthcare provider should recommend taking part in group exercise activities with people that share similar characteristics with the patient. | 11. Group exercise activities with people presenting similar risk factors can help motivate the patient to engage in regular physical activity. | 11. Group exercise activities with people presenting similar risk factors could motivate the patient to engage in regular physical activity. |
| **13. Patients at risk of HYPOGLYCEMIA should:** |  |  |
| 13.2 Take slow-release carbohydrates with them during the exercise session. | 13.2 Slow-release carbohydrates should be taken before exercise. | 13.2. Slow-absorbing carbohydrates should be consumed before any prolonged exercise. |
| 13.3 Drink liquids during exercise. | 13.3 It is important to drink plenty of liquid during exercise, to promote hydration. |  |
| **SPECIFIC RECOMMENDATIONS PRIOR TO PHYSICAL ACTIVITY FOR PEOPLE WITH IWGDF RISK 1, 2, AND 3:** | | |
| **18. Patients with IWGDF 2 or 3 should inspect their feet:** |  |  |
| 18.2 During exercise. | 18.2 Any prolonged exercise session should be interrupted periodically to test for possible injury. | 18.2. During any prolonged physical exercise (exceeding one hour), it is advisable to examine the feet (without socks) for possible injury. |
| 18.5 Foot inspections should consider sensations described by the patient, such as pain, paresthesia (pins and needles), or itching. | 18.5 Sensations such as pain, paraesthesia or itching should be taken into account. |  |
| **19. In case of NEUROPATHY, patients should consult a podiatrist for:** |  |  |
| 19.2 A prescription for biomechanical footwear with a flexible, semirigid sole at IWGDF 2. | 19.2 At IWGDF 2, biomechanical footwear should be prescribed. |  |
| 19.3 A recommendation on the use of biomechanical footwear with a rigid sole at IWGDF 3. | 19.3 At IWGDF 3, biomechanical and/or custom-made footwear should be prescribed. |  |
| **SPECIFIC RECOMMENDATIONS PRIOR TO PHYSICAL ACTIVITY FOR PEOPLE WITH IWGDF RISK 3:** | | |
| 20. Patients who have had an active ulceration can begin putting weight on the foot 15 days after epithelialization is finished, without temporary offloading. | 20. After active ulceration, the patient may begin putting weight on the foot 15 days after epithelialisation is complete, without temporary offloading (using felt, post-surgical shoes or other devices) as long as there is a definitive offloading mechanism (i.e. an insole). | 20. If the patient has suffered active ulceration, the start of support (using a felt insole, post-surgical shoes or other devices) may be recommended, provided this is at least 15 days after full epithelialisation with no temporary discharge, and that a definitive orthotic support device is available. Any such support should always be provided gradually and progressively. |
| **21. If the ulcer had a plantar location and has completely healed (the wound has not opened for 15 days after epithelialization), before beginning exercise, the patient should:** |  |  |
| 21.2 Use biomechanical footwear with a rigid sole. | 21.2 Patients should use biomechanical footwear and/or footwear adapted to individual characteristics (such as high toe or special width). |  |
| **TYPE OF EXERCISE: INTENSITY, DURATION, FREQUENCY, PROGRESSION:** General recommendations for people at any IWGDF risk (categories 0–3) regarding the type of exercise: intensity, duration, frequency, progression. | | |
| **22. When the patient spends a long period of time in a sedentary state, for every 30 min, they should spend at least 3 min walking around or stretching their legs and arms, regardless of whether they are regularly exercising.** | 22. Prolonged physical inactivity should be avoided. Every 30 minutes during a sedentary state, the patient should spend at least three minutes walking around, stretching their arms and legs, regardless of whether regular physical exercise is also taken. |  |
| **24. In patients receiving rehabilitation treatment, telemonitoring of physical activity can improve adherence to treatment in patients with all categories of foot risk.** | 24. In patients receiving rehabilitation treatment, telemonitoring facilitates the control of adherence to the exercise programme. |  |
| **25. In people with diabetes, telemonitoring of physical activity can improve adherence to treatment in patients with all categories of foot risk.** | 25. The use of telemonitoring can improve patients’ adherence to physical exercise programmes, whatever the degree of foot risk. |  |
| **26. All programmed sessions of physical activity should include:** |  |  |
| 26.4 On finishing the exercise, stretches in at least the muscles worked, even if the patient does not do any general stretching. | 26.4 On finishing the exercise programme, even if general stretching is not performed, at least the muscles worked in that session should be worked. | 26.4 On finishing the exercise programme, even if general stretching is not performed, at least the muscles worked in that session should be STRETCHED. |
| **27. Patients with neuropathy (IWGDF 1, 2 and 3) should:** |  |  |
| 27.1 Do exercises to improve static balance: one-legged poses, adapted to the patient’s individual characteristics and exercise programs, and performed in tandem on 2 or 3 non-consecutive days a week. | 27.1 Do exercises to improve static balance. |  |
| 27.2 Do exercises to improve dynamic balance: walking several meters in tandem, laterally, and backwards, on 2 or 3 non-consecutive days a week, with activities adapted to the patient’s individual characteristics and exercise programs. | 27.2 Do exercises to improve dynamic balance. |  |
| **28. In patients with neuropathy, rehabilitation exercises are advisable to improve or limit the progression of the neuropathy, regardless of IWGDF risk** | 28. In patients with neuropathy, rehabilitation exercises are advisable to improve or limit the progression of the motor neuropathy, regardless of IWGDF risk | 28. If the patient has suffered motor neuropathy, regardless of the degree of risk, rehabilitation exercises should be performed to limit the progression of the deformities. |
| **SPECIFIC RECOMMENDATIONS FOR PATIENTS WITH IWGDF 0, ACCORDING TO THE TYPE OF EXERCISE: INTENSITY, DURATION, FREQUENCY, PROGRESSION.** | | |
| **31. In the absence of any cardiovascular alterations, the patient should, (at least):** |  |  |
| 31.3 Do aerobic exercise at least 3 days/week, without going 2 consecutive days without exercise. | 31.3 Aerobic exercise should be performed at least three days a week, without leaving intervals of two consecutive days without physical activity. | 31.3 Aerobic exercise should be performed at least five days a week, without leaving intervals of two consecutive days without physical activity. |
| 33.4 In case of cycling, use an offloading device on the pedal to distribute plantar stress if the patient has a specific area of hyperkeratosis. |  | 33.4. If the patient rides a bicycle, in addition to recommending a specific orthosis within the footwear according to the biomechanical alteration presented, a discharge device could be added to the pedal area to help redistribute the pressure if this area presents hyperkeratosis. |
| **SPECIFIC RECOMMENDATIONS FOR PATIENTS WITH IWGDF 1. ACCORDING TO THE TYPE OF EXERCISE: INTENSITY, DURATION, FREQUENCY, PROGRESSION.** | | |
| **33. Patients with peripheral neuropathy should:** |  |  |
| 33.1 Walk (moderate exercise) for 1 h/3 times a week, beginning with 30 min sessions. | 33.1 Patients should walk (at a moderate pace) for one hour, three times a week, starting with 30 minutes per session and progressively increasing the duration. |  |
| 33.2 Do strength training: resisted active movements (using a band), working the ankle (dorsi and plantar flexion), the forefoot (inversion-eversion) and the toes (flexion-extension, abduction-adduction) at least twice a week. | 33.2 Muscle strengthening exercises, with active movements against resistance, should be performed at least twice a week. |  |
| 33.4 In case of cycling, use an offloading device on the pedal to distribute plantar stress if the patient has a specific area of hyperkeratosis. | 33.4 When cycling, subject to a biomechanical study, an offloading device should be placed on the pedal to distribute plantar pressure, if the patient has a specific area of hyperkeratosis. |  |
| **SPECIFIC RECOMMENDATIONS FOR PATIENTS WITH IWGDF 2, ACCORDING TO THE TYPE OF EXERCISE: INTENSITY, DURATION, FREQUENCY, PROGRESSION.** | | |
| **35. Patients with IWGDF 2, neuropathy and foot deformity should:** |  | |
| 35.1 Do low-intensity aerobic exercise, walking for 15-20 min 3 times a week or every other day. | 35.1 Low-intensity aerobic exercise is recommended, initially for 5-10 minutes and progressively increasing to a duration of 25-30 minutes, performed three times a week or on alternate days. | 35.1 Low-intensity aerobic exercise is recommended, initially for 5-10 minutes and progressively increasing to a duration of 25-30 minutes per day during the week on alternate days. |
| 35.2 In case of cycling, use an offloading device on the pedal to distribute plantar stress if the patient has a specific area of hyperkeratosis. | 35.2 When cycling, subject to a biomechanical study, an offloading device should be placed on the pedal to distribute plantar pressure, if the patient has a specific area of hyperkeratosis. | 35.2. If the patient rides a bicycle, in addition to recommending a specific orthosis within the footwear according to the biomechanical alteration presented, a discharge device could be added to the pedal area to help redistribute the pressure if this area presents hyperkeratosis. |
| 35.3 Add range-of-motion exercises: passive movements to the extent possible in the ankle joints (dorsi and plantar flexion), the forefoot (inversion-eversion) and the toes (flexion-extension, abduction-adduction) at least twice a week. | 35.3 Add range-of-motion exercises: passive movements to the extent possible in the ankle joints (dorsi and plantar flexion), the forefoot (inversion-eversion) and the toes (flexion-extension, abduction-adduction) at least twice a week. |  |
| **36. Patients with IWGDF 2, neuropathy and foot deformity should go swimming or participate in other aquatic activities that do not put pressure on the foot, 2 or 3 times a week.** | 36. Patients with IWGDF 2, neuropathy and foot deformity should go swimming or participate in other aquatic activities that do not put pressure on the foot, 2 or 3 times a week. | 36. For patients with Grade 2 foot risk, with neuropathy and deformity, the aerobic exercise of walking can be replaced by aquatic activities, producing no pressure on the foot, 2-3 times a week. These patients should always use appropriate footwear and maintain good foot hygiene |
| **37. Patients with IWGDF 2, neuropathy and foot deformity should use a stationary pedal exerciser in a seated, strain-free position for 15 min to 20 min a day, at least 4 days a week, without going 2 consecutive days without physical exercise.** | 37. Patients with IWGDF 2 and presenting neuropathy and deformity should use a stationary pedal exerciser in a seated position, with little pressure on the foot, for 25-30 minutes/day, at least four days a week, without leaving intervals of two consecutive days without physical activity. | 37. In patients with Grade 2 foot risk, with neuropathy and deformity, and high risk of ulceration, any exercise taken should exert little pressure on the foot, such as the use of static pedals in a sitting position. This exercise should be for 25-30 minutes daily, at least four days a week, and never letting more than two consecutive days pass without taking physical exercise. |
| **SPECIFIC RECOMMENDATIONS FOR PATIENTS WITH IWGDF 3, ACCORDING TO THE TYPE OF EXERCISE: INTENSITY, DURATION, FREQUENCY, PROGRESSION.** | | |
| **41. Patients with severe PAD (with lesions) should:** |  |  |
| 41.2 Work on ankle mobility, plantar flexion, dorsiflexion, inversion, eversion, circumduction, and dorsi and plantar flexion of the toes at least 3 times a week or every other day. | 41.2 Exercises to improve ankle mobility, plantar flexion, dorsal flexion, inversion, eversion, circumduction and plantar and dorsal flexion of the toes should be performed three times per week or every other day, provided it does not provoke pain or aggravate the injury. |  |
| **42. Patients with IWGDF 3 and a recent (< 15 days) history of ulceration should:** |  |  |
| 42.1 Begin by doing at least 10 min of daily activity with a technical aid (cane, crutch) and gradually progress over the following 15 days if their condition allows it. | 42.1 Initially, the patient should perform at least ten minutes’ activity per day wearing a plantar orthosis and with technical assistance (using a cane or crutch), and gradually increase the duration over the next 15 days, if possible. | 42.1. The activities of daily life should be started, with at least 10 minutes’ activity every day, using a plantar orthosis and technical assistance (such as a cane or crutch) and gradually progressing over the next 15 days, according to the patient’s physical condition. |
| 42.2 Begin by doing at least 10 min of daily activity and gradually progress over the following 15 days if their condition allows it. | 42.2 Initially, the patient should perform at least ten minutes’ activity per day wearing a plantar orthosis and gradually increase the duration over the next 15 days, if possible. |  |
| **43. At ONE MONTH OF HEALING, patients with IWGDF 3 should:** |  |  |
| 43.1 Begin walking 15 min to 20 min a day, 3 days a week, at a low intensity. | 43.1 Initially, the patient should walk for 15-20 minutes, three times a week, at low intensity, and always with a plantar orthosis. | 43.1. At first, the patient should walk gently for 15-20 minutes, three times a week, always using a plantar orthosis and wearing appropriate biomechanical footwear. |
| 43.2 Go swimming or do aquatic activities that do not put pressure on the foot, 2 or 3 times a week. |  | 43.2. Swimming or other aquatic activity, exerting no pressure on the foot, is recommended, 2-3 times a week, wearing appropriate footwear and practising appropriate measures of foot hygiene |
| 43.3 Use a stationary pedal exerciser in a seated position, starting with 15 min to 20 min a day, 3 days a week or every other day. | 43.3 Initially, the patient should use a stationary pedal exerciser in a seated position for 15-20 minutes per day, three times a week or every other day, and always with a plantar orthosis. |  |
| 43.4 Do yoga or other stretching and balance exercises, 2 to 3 times a week. | 43.4 Yoga, or stretching and balance exercises, should be performed two or three times a week, and always with a plantar orthosis. |  |
| **RECOMMENDATIONS DURING EXERCISE : SPECIFIC RECOMMENDATIONS FOR PATIENTS WITH IWGDF 1, 2, OR 3** | | |
| **45. Patients with an at-risk foot, neuropathy, and/or deformity should walk on shock-absorbing surfaces (grass, clay), without excluding other types of surfaces.** | 45. Patients with an at-risk foot, neuropathy and/or deformity should walk on smooth, shock-absorbing surfaces, such as grass or sand. |  |
| **46. The use of a cane should be considered in patients with neuropathy and foot deformity, in case of important alterations in proprioception** | 46 Patients with neuropathy and foot deformity should consider using an assistive device, such as a cane or crutch, if their balance is significantly disturbed. |  |
| **49. Healthcare providers should follow up patients with IWGDF 2 and 3 every month to monitor the recommended exercise regimen and modify it according to the patient’s evolution.** | 49. Healthcare providers should follow up patients with IWGDF 2 and 3 every month to monitor the recommended exercise regimen and modify it according to the patient’s evolution. |  |
| **50. All patients with neuropathy who use custom-made soles should be followed at 15 days following their use.** | 50. Any patient with diabetes and neuropathy, or who uses a custom-made plantar orthosis, should be examined 15 days after starting physical exercise by the healthcare provider. This examination should be repeated periodically to detect any wear or irregularities in the material after its use in physical exercise. |  |

| **CAPTION FOR TABLE** | |
| --- | --- |
| **Bold tipe** | Main statement |
| Table cell black | Title thematic blocks |

| **Supplementary material 2 – S.3.** Results of the 2ND round of the Delphi survey. |  |  |  |
| --- | --- | --- | --- |
| **General recommendations prior to commencing physical activity.**  General recommendations for people at any IWGDF risk (categories 0–3) prior to commencing physical activity. |  |  |  |
| **1.The patient themselves should inspect both feet before beginning physical activity, checking for:** | **Agree (%)** | **Disagree**  **(%)** | **Neutral**  **(%)** |
| 1.2 Ensure proper toenail cutting, with a straight cut | 75.8 | 10.3 | 13.8 |
| **2. An initial examination of the patient’s general condition should be undertaken, including an assessment of:** |  |  |  |
| 2.1 Body Mass Index (BMI). Recommended activity levels should take into account the presence of obesity | 86.2 | 3.4 | 10.3 |
| 2.6 The presence of uncontrolled retinopathy or renal impairment may influence the type of exercise recommended. | 100 |  |  |
| **4. People under treatment with insulin and/or sulfonylureas:** |  |  |  |
| 4.2 Glucose levels should be monitored during exercise, and more frequently for high-risk patients being treated with insulin. | 82.7 | 0 | 17.2 |
| 4.4 If necessary, dietary and/or pharmacological adjustments should be recommended by the healthcare provider, and a glycogen kit should be available. | 86.2 | 0 | 13.8 |
| **5. The presence of keratotic lesions or excessive dryness on different areas of the foot:** |  |  |  |
| 5.1 These conditions should be treated before establishing the intensity and duration of the exercise recommended. | 86.2 | 10.3 | 3.4 |
| 5.2 These conditions should be treated before establishing the type of exercise recommended | 93.1 | 6.9 | 0 |
| **6. The type of sock recommended should be conditioned by:** |  |  |  |
| 6.2 Socks should suit the physical activity to be performed. They should be made of cotton, light coloured to make any bleeding easily visible, seamless and with no tight-fitting elastic. | 69 | 10.3 | 20.7 |
| **7. In the case of PAD (peripheral artery disease), the patient should use:** |  |  |  |
| 7.1 Socks made of natural fibers that help regulate foot temperature. | 58.3 | 3.4 | 37.9 |
| 7.3 Socks should contain a blend of synthetic fibres to wick moisture away from the foot.. | 20.7 | 17.2 | 62.1 |
| 7.4 Socks should facilitate temperature monitoring in order to indicate possible injury during exercise. | 13.8 | 31 | 55.2 |
| 7.5 Socks should incorporate antimicrobial compounds to reduce the risk of foot infections. | 14 | 41 | 45 |
| **8. In case of NEUROPATHY, patients should use:** |  |  |  |
| 8.1 Socks made of natural fibers. | 65.5 | 3.4 | 31 |
| 8.2 Socks made with a blend of synthetic fibers that wick moisture away from the foot. | 10.3 | 27.6 | 62.1 |
| 8.4 Socks should facilitate temperature monitoring in order to indicate possible injury during exercise. | 10.3 | 27.3 | 62.1 |
| 8.5 Socks should incorporate antimicrobial compounds to reduce the risk of foot infections. | 17.2 | 41.4 | 41.4 |
|  | **Agree (%)** | **Disagree**  **(%)** | **Neutral**  **(%)** |
| 9. A professional should always be consulted about the type of footwear before the patient begins an exercise regimen. | 93.1 | 3.4 | 3.4 |
| **11. Group exercise activities with people presenting similar risk factors can help motivate the patient to engage in regular physical activity.** | 76 | 0 | 24 |
| **12. The use of a smart watch or mobile apps during exercise is advised in patients with HIGH CARDIOVASCULAR RISK in order to monitor:** |  |  |  |
| 12.2 Blood pressure. | 82.8 | 6.9 | 10.3 |
| 12.3 Intensity of physical activity. | 89.7 | 0 | 10.3 |
| 12.4 Type and duration of physical activity. | 93.1 | 0 | 6.9 |
| 12.5 Blood oxygen level, with accredited pulse oximeter. | 24 | 31 | 45 |
| **13. Patients at risk of HYPOGLYCEMIA should:** |  |  |  |
| 13.2 Slow-release carbohydrates should be taken before exercise. | 48 | 3 | 48 |
| 13.3 It is important to drink plenty of liquid during exercise, to promote hydration. | 93.1 | 3.4 | 3.4 |
| **SPECIFIC RECOMMENDATIONS PRIOR TO PHYSICAL ACTIVITY FOR PEOPLE WITH IWGDF RISK 1, 2, AND 3:** |  |  |  |
| **18. Patients with IWGDF 2 or 3 should inspect their feet:** |  |  |  |
| 18.2 Any prolonged exercise session should be interrupted periodically to test for possible injury. | 62.1 | 13.8 | 24.1 |
| 18.5 Sensations such as pain, paraesthesia or itching should be taken into account. | 96.6 | 3.4 | 0 |
| **19. In case of NEUROPATHY, patients should consult a podiatrist for:** |  |  |  |
| 19.2 At IWGDF 2, biomechanical footwear should be prescribed. | 89.7 | 6.9 | 3.4 |
| 19.3 IWGDF 3, biomechanical and/or custom-made footwear should be prescribed. | 96.6 | 3.4 | 0 |
| **SPECIFIC RECOMMENDATIONS PRIOR TO PHYSICAL ACTIVITY FOR PEOPLE WITH IWGDF RISK 3:** |  |  |  |
| **20. After active ulceration, the patient may begin putting weight on the foot 15 days after epithelialisation is complete, without temporary offloading (using felt, post-surgical shoes or other devices) as long as there is a definitive offloading mechanism (i.e. an insole).** | 37.9 | 34.5 | 27.6 |
| **21. If the ulcer had a plantar location and has completely healed (the wound has not opened for 15 days after epithelialization), before beginning exercise, the patient should:** |  |  |  |
| 21.2 Patients should use biomechanical footwear and/or footwear adapted to individual characteristics (such as high toe or special width). | 96.6 | 0 | 3.4 |
| General recommendations for people at any IWGDF risk (categories 0–3) regarding the type of exercise: intensity, duration, frequency, progression. |  |  |  |
|  | **Agree (%)** | **Disagree**  **(%)** | **Neutral**  **(%)** |
| **22. Prolonged physical inactivity should be avoided. Every 30 minutes during a sedentary state, the patient should spend at least three minutes walking around, stretching their arms and legs, regardless of whether regular physical exercise is also taken.** | 79.3 | 0 | 20.7 |
| **24. In patients receiving rehabilitation treatment, telemonitoring facilitates the control of adherence to the exercise programme.** | 86.2 | 0 | 13.8 |
| 25. The use of telemonitoring can improve patients’ adherence to physical exercise programmes, whatever the degree of foot risk. | 86.2 | 0 | 13.8 |
| **26. All programmed sessions of physical activity should include:** |  |  |  |
| 26.3 On finishing the exercise, a cool-down of at least 5 min of slow walking. | 82.8 | 6.9 | 10.3 |
| 26.4 On finishing the exercise programme, even if general stretching is not performed, at least the muscles worked in that session should be worked. | 79.3 | 10.3 | 10.3 |
| **27. Patients with neuropathy (IWGDF 1, 2 and 3) should:** |  |  |  |
| 27.1 Do exercises to improve static balance. | 82.8 | 3.4 | 13.8 |
| 27.2 Do exercises to improve dynamic balance. | 86.2 | 3.4 | 10.3 |
| **28. In patients with neuropathy, rehabilitation exercises are advisable to improve or limit the progression of the motor neuropathy, regardless of IWGDF risk.** | 69 | 10.3 | 20.7 |
| **29. In patients with mild to moderate PAD (IWGDF 1, 2, or 3), the healthcare provider should consider adding specific exercises to improve vascular function** | 86.2 | 6.9 | 6.9 |
| **SPECIFIC RECOMMENDATIONS FOR PATIENTS WITH IWGDF 0, ACCORDING TO THE TYPE OF EXERCISE: INTENSITY, DURATION, FREQUENCY, PROGRESSION.** |  |  |  |
| **31. In the absence of any cardiovascular alterations, the patient should, (at least):** |  |  |  |
| 31.2 Gradually increase daily moderate aerobic activity from 30 min/day to 1 h/day. | 75.9 | 6.9 | 17.2 |
| 31.3 Aerobic exercise should be performed at least three days a week, without leaving intervals of two consecutive days without physical activity. | 72.4 | 3.4 | 24.1 |
| 32. Exercise activities should include strength training: from passive movements of the ankle joint to active resistance (using a band) to work the ankle (dorsi and plantar flexion), the forefoot (inversion-eversion) and the toes (flexion-extension, abduction-adduction) at least twice a week. | 82.8 | 3.4 | 13.8 |
| **SPECIFIC RECOMMENDATIONS FOR PATIENTS WITH IWGDF 1. ACCORDING TO THE TYPE OF EXERCISE: INTENSITY, DURATION, FREQUENCY, PROGRESSION.** | **Agree (%)** | **Disagree**  **(%)** | **Neutral**  **(%)** |
| **33. Patients with peripheral neuropathy should:** |  |  |  |
| 33.1 Patients should walk (at a moderate pace) for one hour, three times a week, starting with 30 minutes per session and progressively increasing the duration. | 82.8 | 13.8 | 3.4 |
| 33.2 Muscle strengthening exercises, with active movements against resistance, should be performed at least twice a week. | 89.7 | 0 | 10.3 |
| 33.3 In case of coexisting obesity, do aerobic activity that does not overload joints or put excessive pressure on the feet (cycling, swimming). | 93.1 | 0 | 6.9 |
| 33.4 When cycling, subject to a biomechanical study, an offloading device should be placed on the pedal to distribute plantar pressure, if the patient has a specific area of hyperkeratosis. | 69 | 6.9 | 24.1 |
| 34. Patients with mild to moderate PAD (Rutherford grade 1 or 2) WITHOUT NEUROPATHY should begin walking at a moderate intensity and gradually increase it based on the onset of pain due to intermittent claudication. | 82.8 | 3.4 | 13.8 |
| **SPECIFIC RECOMMENDATIONS FOR PATIENTS WITH IWGDF 2, ACCORDING TO THE TYPE OF EXERCISE: INTENSITY, DURATION, FREQUENCY, PROGRESSION.** |  |  |  |
| **35. Patients with IWGDF 2, neuropathy and foot deformity should:** |  |  |  |
| 35.1 Low-intensity aerobic exercise is recommended, initially for 5-10 minutes and progressively increasing to a duration of 25-30 minutes, performed three times a week or on alternate days. | 75.1 | 3.4 | 20.7 |
| 35.2 When cycling, subject to a biomechanical study, an offloading device should be placed on the pedal to distribute plantar pressure, if the patient has a specific area of hyperkeratosis. | 75.9 | 13.8 | 10 |
| 35.3 Add range-of-motion exercises: passive movements to the extent possible in the ankle joints (dorsi and plantar flexion), the forefoot (inversion-eversion) and the toes (flexion-extension, abduction-adduction) at least twice a week. | 93.1 | 0 | 6.9 |
| **36. Patients with IWGDF 2, neuropathy and foot deformity should go swimming or participate in other aquatic activities that do not put pressure on the foot, 2 or 3 times a week.** | 72.4 | 0 | 27.6 |
| **37. Patients with IWGDF 2 and presenting neuropathy and deformity should use a stationary pedal exerciser in a seated position, with little pressure on the foot, for 25-30 minutes/day, at least four days a week, without leaving intervals of two consecutive days without physical activity.** | 55.2 | 3.4 | 41.4 |
| **SPECIFIC RECOMMENDATIONS FOR PATIENTS WITH IWGDF 3, ACCORDING TO THE TYPE OF EXERCISE: INTENSITY, DURATION, FREQUENCY, PROGRESSION.** | **Agree (%)** | **Disagree**  **(%)** | **Neutral**  **(%)** |
| **40. Patients with IWGDF 3 and an open ulcer without PAD should:** |  |  |  |
| 40.1 Stretching and strength-training exercises should be performed, in a seated or supine position, according to the patient’s characteristics and the location of the ulcer. | 96.6 | 0 | 3.4 |
| 40.2 Work on ankle mobility, plantar flexion, dorsiflexion, inversion, eversion, circumduction, and dorsi and plantar flexion of the toes at least 3 times a week or every other day, adapting all activities to the patient’s characteristics. | 82.8 | 3.4 | 13.8 |
| 40.3 In each set of mobility exercises, do at least 5 to 10 exercises with 10 to 15 repetitions each, 3 times a week or every other day, adapting all activities to the patient’s characteristics. | 65.5 | 0 | 34.5 |
| **41. Patients with severe PAD (with lesions) should:** |  |  |  |
| 41.2 Exercises to improve ankle mobility, plantar flexion, dorsal flexion, inversion, eversion, circumduction and plantar and dorsal flexion of the toes should be performed three times per week or every other day, provided it does not provoke pain or aggravate the injury. | 93.1 | 0 | 6.9 |
| **42. Patients with IWGDF 3 and a recent (< 15 days) history of ulceration should:** |  |  |  |
| 42.1 Initially, the patient should perform at least ten minutes’ activity per day wearing a plantar orthosis and with technical assistance (using a cane or crutch), and gradually increase the duration over the next 15 days, if possible. | 72.4 | 6.9 | 20.7 |
| 42.2 Initially, the patient should perform at least ten minutes’ activity per day wearing a plantar orthosis and gradually increase the duration over the next 15 days, if possible. | 86.2 | 3.4 | 10.3 |
| **43. At ONE MONTH OF HEALING, patients with IWGDF 3 should:** |  |  |  |
| 43.1 Initially, the patient should walk for 15-20 minutes, three times a week, at low intensity, and always with a plantar orthosis. | 72.4 | 6.9 | 20.7 |
| 43.2 Go swimming or do aquatic activities that do not put pressure on the foot, 2 or 3 times a week. | 65.5 | 0 | 34.5 |
| 43.3 Initially, the patient should use a stationary pedal exerciser in a seated position for 15-20 minutes per day, three times a week or every other day, and always with a plantar orthosis. | 58.6 | 6.9 | 34.5 |
| 43.4 Yoga, or stretching and balance exercises, should be performed two or three times a week, and always with a plantar orthosis. | 55.2 | 3.4 | 41.4 |
| RECOMMENDATIONS DURING EXERCISE:  Specific recommendations for patients with IWGDF 1, 2, or 3 | **Agree (%)** | **Disagree**  **(%)** | **Neutral**  **(%)** |
| **45. Patients with an at-risk foot, neuropathy and/or deformity should walk on smooth, shock-absorbing surfaces, such as grass or sand.** | 65.5 | 17.2 | 17.2 |
| **46. Patients with neuropathy and foot deformity should consider using an assistive device, such as a cane or crutch, if their balance is significantly disturbed..** | 96.6 | 0 | 3.4 |
| **47. If the patient has a history of ulcers, they should use monitoring devices with alarms to control temperature and/or foot pressure during the activity.** | 41.4 | 17.2 | 41.4 |
| RECOMMENDATIONS FOLLOWING EXERCISE:  General recommendations following exercise for patients with all categories of foot risk. |  |  |  |
| **49. Healthcare providers should follow up patients with IWGDF 2 and 3 every month to monitor the recommended exercise regimen and modify it according to the patient’s evolution.** | 93.1 | 3.4 | 3.4 |
| **50. Any patient with diabetes and neuropathy, or who uses a custom-made plantar orthosis, should be examined 15 days after starting physical exercise by the healthcare provider. This examination should be repeated periodically to detect any wear or irregularities in the material after its use in physical exercise.** | 93.1 | 0 | 6.9 |

| **Supplementary material 2 – S.4.** Results of the 3RD round of the Delphi survey. | | | |
| --- | --- | --- | --- |
| **General recommendations prior to commencing physical activity:**  General recommendations for people at any IWGDF risk (categories 0–3) prior to commencing physical activity. | | | |
| **1. The patient themselves should inspect both feet before beginning physical activity, checking for:** | **Agree**  **(%)** | **Disagree**  **(%)** | **Neutral**  **(%)** |
| 1.2. Cut toenails appropriately, according to their morphology. | 78.6 | 3.6 | 17.9 |
| **6. The type of sock recommended should be conditioned by:** |  |  |  |
| 6.2. The type of sock to be recommended should be specific, according to the physical activity to be carried out, but preferably light-coloured to visualise any bleeding more easily, clean, seamless and with no tight-fitting elastics. | 89.3 | 3.6 | 7.1 |
| **7. In the case of PAD (peripheral artery disease), the patient should use:** | | | |
| 7.1. Socks that help maintain the temperature of the foot should be recommended. | 67.9 | 10.7 | 21.4 |
| 7.3 Socks should contain a blend of synthetic fibres to wick moisture away from the foot. | 21.4 | 42.9 | 35.7 |
| 7.4. Socks providing continuous temperature control should be recommended, to reveal possible vascular injuries during exercise. | 7.1 | 57.1 | 35.7 |
| 7.5. Socks that incorporate antibacterial compounds should be recommended, to reduce the risk of infection in patients with vascular lesions. | 17.9 | 60.7 | 21.4 |
| **8. In case of NEUROPATHY, patients should use:** | | | |
| 8.1 Socks made of natural fibers. | 67.9 | 7.1 | 25 |
| 8.2 Socks made with a blend of synthetic fibers that wick moisture away from the foot. | 17.9 | 50 | 32.1 |
| 8.4 Socks should facilitate temperature monitoring in order to indicate possible injury during exercise. | 14.3 | 60.7 | 25 |
| 8.5 Socks should incorporate antimicrobial compounds to reduce the risk of foot infections. | 10.7 | 75 | 14.3 |
| **11. Group exercise activities with people presenting similar risk factors can help motivate the patient to engage in regular physical activity.** | 89.3 | 3.6 | 7.1 |
| **12. The use of a smart watch or mobile apps during exercise is advised in patients with HIGH CARDIOVASCULAR RISK in order to monitor:** |  |  |  |
| 12.5 Blood oxygen level, with accredited pulse oximeter. | 35.7 | 35.7 | 28.6 |
| **13. Patients at risk of HYPOGLYCEMIA should:** |  |  |  |
| 13.2. Slow-absorbing carbohydrates should be consumed before any prolonged exercise. | 64.3 | 7.1 | 28.6 |
| **SPECIFIC RECOMMENDATIONS PRIOR TO PHYSICAL ACTIVITY FOR PEOPLE WITH IWGDF RISK 1, 2, AND 3:** | | | |
| **18. Patients with IWGDF 2 or 3 should inspect their feet:** | **Agree**  **(%)** | **Disagree**  **(%)** | **Neutral**  **(%)** |
| 18.2. During any prolonged physical exercise (exceeding one hour), it is advisable to examine the feet (without socks) for possible injury. | 89.3 | 7.1 | 3.6 |
| **SPECIFIC RECOMMENDATIONS PRIOR TO PHYSICAL ACTIVITY FOR PEOPLE WITH IWGDF RISK 3:** |  |  |  |
| **20. If the patient has suffered active ulceration, the start of support (using a felt insole, post-surgical shoes or other devices) may be recommended, provided this is at least 15 days after full epithelialisation with no temporary discharge, and that a definitive orthotic support device is available. Any such support should always be provided gradually and progressively.** | 57.1 | 17.9 | 25 |
| **Type of exercise: intensity, duration, frequency, progression:**  **General recommendations for people at any IWGDF risk (categories 0–3) regarding the type of exercise: intensity, duration, frequency, progression.** | | | |
| **22. Prolonged physical inactivity should be avoided. Every 30 minutes during a sedentary state, the patient should spend at least three minutes walking around, stretching their arms and legs, regardless of whether regular physical exercise is also taken.** | 71.4 | 0 | 28.6 |
| **26. All programmed sessions of physical activity should include:** | | | |
| 26.4 On finishing the exercise programme, even if general stretching is not performed, at least the muscles worked in that session should be STRETCHED. | 67.9 | 7.1 | 25 |
| **28. If the patient has suffered motor neuropathy, regardless of the degree of risk, rehabilitation exercises should be performed to limit the progression of the deformities.** | 85.7 | 3.6 | 10.7 |
| **SPECIFIC RECOMMENDATIONS FOR PATIENTS WITH IWGDF 0, ACCORDING TO THE TYPE OF EXERCISE: INTENSITY, DURATION, FREQUENCY, PROGRESSION.V** | | | |
| **31. In the absence of any cardiovascular alterations, the patient should, (at least):** | | | |
| 31.2. Each exercise session should consist of 30-60 minutes of aerobic exercise. | 82.1 | 0 | 17.9 |
| 31.3 Aerobic exercise should be performed at least five days a week, without leaving intervals of two consecutive days without physical activity. | 67.9 | 3.6 | 28.6 |
| **SPECIFIC RECOMMENDATIONS FOR PATIENTS WITH IWGDF 1. ACCORDING TO THE TYPE OF EXERCISE: INTENSITY, DURATION, FREQUENCY, PROGRESSION.** |  |  |  |
| **33. Patients with peripheral neuropathy should:** |  |  |  |
| 33.4. If the patient rides a bicycle, in addition to recommending a specific orthosis within the footwear according to the biomechanical alteration presented, a discharge device could be added to the pedal area to help redistribute the pressure if this area presents hyperkeratosis. | 82.1 | 3.6 | 14.3 |
| **SPECIFIC RECOMMENDATIONS FOR PATIENTS WITH IWGDF 2, ACCORDING TO THE TYPE OF EXERCISE: INTENSITY, DURATION, FREQUENCY, PROGRESSION.** | **Agree**  **(%)** | **Disagree**  **(%)** | **Neutral**  **(%)** |
| **35. Patients with IWGDF 2, neuropathy and foot deformity should:** |  |  |  |
| 35.1 Low-intensity aerobic exercise is recommended, initially for 5-10 minutes and progressively increasing to a duration of 25-30 minutes per day during the week on alternate days. | 85.7 | 3.6 | 10.7 |
| 35.2. If the patient rides a bicycle, in addition to recommending a specific orthosis within the footwear according to the biomechanical alteration presented, a discharge device could be added to the pedal area to help redistribute the pressure if this area presents hyperkeratosis. | 82.1 | 3.6 | 14.3 |
| **36. For patients with Grade 2 foot risk, with neuropathy and deformity, the aerobic exercise of walking can be replaced by aquatic activities, producing no pressure on the foot, 2-3 times a week. These patients should always use appropriate footwear and maintain good foot hygiene.** | 85.7 | 10.7 | 3.6 |
| **SPECIFIC RECOMMENDATIONS FOR PATIENTS WITH IWGDF 2, ACCORDING TO THE TYPE OF EXERCISE: INTENSITY, DURATION, FREQUENCY, PROGRESSION.** | | | |
| **37. In patients with Grade 2 foot risk, with neuropathy and deformity, and high risk of ulceration, any exercise taken should exert little pressure on the foot, such as the use of static pedals in a sitting position. This exercise should be for 25-30 minutes daily, at least four days a week, and never letting more than two consecutive days pass without taking physical exercise**. | 64.3 | 17.9 | 17.9 |
| **SPECIFIC RECOMMENDATIONS FOR PATIENTS WITH IWGDF 3, ACCORDING TO THE TYPE OF EXERCISE: INTENSITY, DURATION, FREQUENCY, PROGRESSION.** | | | |
| **40. Patients with IWGDF 3 and an open ulcer without PAD should:** |  |  |  |
| 40.3 In each set of mobility exercises, do at least 5 to 10 exercises with 10 to 15 repetitions each, 3 times a week or every other day, adapting all activities to the patient’s characteristics. | 50 | 10.7 | 39.3 |
| **42. Patients with IWGDF 3 and a recent (< 15 days) history of ulceration should:** |  |  |  |
| 42.1. The activities of daily life should be started, with at least 10 minutes’ activity every day, using a plantar orthosis and technical assistance (such as a cane or crutch) and gradually progressing over the next 15 days, according to the patient’s physical condition. | 82.1 | 7.1 | 10.7 |
| **43. At ONE MONTH OF HEALING, patients with IWGDF 3 should:** | | | |
| 43.1. At first, the patient should walk gently for 15-20 minutes, three times a week, always using a plantar orthosis and wearing appropriate biomechanical footwear. | 78.6 | 7.1 | 14.3 |
| 43.2. Swimming or other aquatic activity, exerting no pressure on the foot, is recommended, 2-3 times a week, wearing appropriate footwear and practising appropriate measures of foot hygiene. | 67.9 | 10.7 | 21.4 |
|  | **Agree**  **(%)** | **Disagree**  **(%)** | **Neutral**  **(%)** |
| 43.3 Initially, the patient should use a stationary pedal exerciser in a seated position for 15-20 minutes per day, three times a week or every other day, and always with a plantar orthosis. | 64.3 | 7.1 | 28.6 |
| 43.4 Yoga, or stretching and balance exercises, should be performed two or three times a week, and always with a plantar orthosis. | 57.1 | 7.1 | 35.7 |
| **Recommendations during exercise:**  Specific recommendations for patients with IWGDF 1, 2, or 3 | | | |
| **45. Patients with an at-risk foot, neuropathy and/or deformity should walk on smooth, shock-absorbing surfaces, such as grass or sand.** | 60.7 | 14.3 | 25 |
| **47. If the patient has a history of ulcers, they should use monitoring devices with alarms to control temperature and/or foot pressure during the activity.** | 28.6 | 28.6 | 42.9 |

| **Supplementary material 2 – S.5.** Items removed from final recommendations due to lack of consensus. | | | |
| --- | --- | --- | --- |
| **General recommendations prior to commencing physical activity:**  **General recommendations for people at any IWGDF risk (categories 0–3) prior to commencing physical activity** | | | |
| **1. The patient themselves should inspect both feet before beginning physical activity, checking for:** | **% Agree** | **% Disagree** | **% Neutral** |
| 1.2. Cut toenails appropriately, according to their morphology. | 78.6% | 3.6% | 17.9% |
| **7. In the case of PAD (peripheral artery disease), the patient should use:** | | | |
| 7.1. Socks that help maintain the temperature of the foot should be recommended. | 67.9% | 10.7% | 21.4% |
| 7.3 Socks should contain a blend of synthetic fibres to wick moisture away from the foot. | 21.4% | 42.9 | 35.7% |
| 7.4. Socks providing continuous temperature control should be recommended, to reveal possible vascular injuries during exercise. | 7.1% | 57.1% | 35.7% |
| 7.5. Socks that incorporate antibacterial compounds should be recommended, to reduce the risk of infection in patients with vascular lesions. | 17.9% | 60.7% | 21.4% |
| **8. In case of NEUROPATHY, patients should use:** | | | |
| 8.1 Socks made of natural fibers. | 67.9% | 7.1% | 25% |
| 8.2 Socks made with a blend of synthetic fibers that wick moisture away from the foot. | 17.9% | 50% | 32.1% |
| 8.4 Socks should facilitate temperature monitoring in order to indicate possible injury during exercise. | 14.3% | 60.7% | 25% |
| 8.5 Socks should incorporate antimicrobial compounds to reduce the risk of foot infections. | 10.7% | 75% | 14.3% |
| **12. The use of a smart watch or mobile apps during exercise is advised in patients with HIGH CARDIOVASCULAR RISK in order to monitor:** |  |  |  |
| 12.5 Blood oxygen level, with accredited pulse oximeter. | 35.7% | 35.7% | 28.6% |
| **13. Patients at risk of HYPOGLYCEMIA should:** |  |  |  |
| 13.2. Slow-absorbing carbohydrates should be consumed before any prolonged exercise. | 64.3% | 7.1% | 28.6% |
| **SPECIFIC RECOMMENDATIONS PRIOR TO PHYSICAL ACTIVITY FOR PEOPLE WITH IWGDF RISK 1, 2, AND 3:** | | | |
| **20. If the patient has suffered active ulceration, the start of support (using a felt insole, post-surgical shoes or other devices) may be recommended, provided this is at least 15 days after full epithelialisation with no temporary discharge, and that a definitive orthotic support device is available. Any such support should always be provided gradually and progressively.** | 57.1% | 17.9% | 25% |
| **TYPE OF EXERCISE: INTENSITY, DURATION, FREQUENCY, PROGRESSION:**  GENERAL RECOMMENDATIONS FOR PEOPLE AT ANY IWGDF RISK (CATEGORIES 0–3) REGARDING THE TYPE OF EXERCISE: INTENSITY, DURATION, FREQUENCY, PROGRESSION. | | | |
| **22. Prolonged physical inactivity should be avoided. Every 30 minutes during a sedentary state, the patient should spend at least three minutes walking around, stretching their arms and legs, regardless of whether regular physical exercise is also taken.** | 71.4% | 0% | 28.6% |
| **26. All programmed sessions of physical activity should include:** | | | |
| 26.4 On finishing the exercise programme, even if general stretching is not performed, at least the muscles worked in that session should be STRETCHED. | 67.9% | 7.1% | 25% |
| **SPECIFIC RECOMMENDATIONS FOR PATIENTS WITH IWGDF 0, ACCORDING TO THE TYPE OF EXERCISE: INTENSITY, DURATION, FREQUENCY, PROGRESSION.V** | | | |
| **31. In the absence of any cardiovascular alterations, the patient should, (at least):** | | | |
| 31.3 Aerobic exercise should be performed at least five days a week, without leaving intervals of two consecutive days without physical activity. | 67.9% | 3.6% | 28.6% |
| **SPECIFIC RECOMMENDATIONS FOR PATIENTS WITH IWGDF 1. ACCORDING TO THE TYPE OF EXERCISE: INTENSITY, DURATION, FREQUENCY, PROGRESSION.** | | | |
| **37. In patients with Grade 2 foot risk, with neuropathy and deformity, and high risk of ulceration, any exercise taken should exert little pressure on the foot, such as the use of static pedals in a sitting position. This exercise should be for 25-30 minutes daily, at least four days a week, and never letting more than two consecutive days pass without taking physical exercise.** | 64.3% | 17.9% | 17.9% |
| **SPECIFIC RECOMMENDATIONS FOR PATIENTS WITH IWGDF 3, ACCORDING TO THE TYPE OF EXERCISE: INTENSITY, DURATION, FREQUENCY, PROGRESSION.** | | | |
| **40. Patients with IWGDF 3 and an open ulcer without PAD should:** |  |  |  |
| 40.3 In each set of mobility exercises, do at least 5 to 10 exercises with 10 to 15 repetitions each, 3 times a week or every other day, adapting all activities to the patient’s characteristics. | 50% | 10.7% | 39.3% |
| **43. At ONE MONTH OF HEALING, patients with IWGDF 3 should:** | | | |
| 43.1. At first, the patient should walk gently for 15-20 minutes, three times a week, always using a plantar orthosis and wearing appropriate biomechanical footwear. | 78.6% | 7.1% | 14.3% |
| 43.2. Swimming or other aquatic activity, exerting no pressure on the foot, is recommended, 2-3 times a week, wearing appropriate footwear and practising appropriate measures of foot hygiene. | 67.9% | 10.7% | 21.4% |
| 43.3 Initially, the patient should use a stationary pedal exerciser in a seated position for 15-20 minutes per day, three times a week or every other day, and always with a plantar orthosis. | 64.3% | 7.1% | 28.6% |
| 43.4 Yoga, or stretching and balance exercises, should be performed two or three times a week, and always with a plantar orthosis. | 57.1% | 7.1% | 35.7% |
| **Recommendations during exercise:**  SPECIFIC RECOMMENDATIONS FOR PATIENTS WITH IWGDF 1, 2, OR 3 | | | |
| **45. Patients with an at-risk foot, neuropathy and/or deformity should walk on smooth, shock-absorbing surfaces, such as grass or sand.** | 60.7% | 14.3% | 25% |
| **47. If the patient has a history of ulcers, they should use monitoring devices with alarms to control temperature and/or foot pressure during the activity.** | 28.6% | 28.6% | 42.9% |
